# Osmotic stress induces long-term biofilm survival in *Liberibacter crescens*

**DOI:** 10.1186/s12866-022-02453-w

**Published:** 2022-02-11

**Authors:** Kaylie A. Padgett-Pagliai, Fernando A. Pagliai, Danilo R. da Silva, Christopher L. Gardner, Graciela L. Lorca, Claudio F. Gonzalez

**Affiliations:** Department of Microbiology and Cell Science, Genetics Institute, Institute of Food and Agricultural Sciences, University of Florida, 2033 Mowry Road, PO Box 103610, Gainesville, FL 32610-3610 USA

**Keywords:** *Liberibacter*, Stress response, RNA-seq, Biofilm, Transcriptome, *Liberibacter crescens*, Osmotic stress, Heat stress, Biofilm

## Abstract

Citrus greening, also known as Huanglongbing (HLB), is a devastating citrus plant disease caused predominantly by *Liberibacter asiaticus*. While nearly all *Liberibacter* species remain uncultured, here we used the culturable *L. crescens* BT-1 as a model to examine physiological changes in response to the variable osmotic conditions and nutrient availability encountered within the citrus host. Similarly, physiological responses to changes in growth temperature and dimethyl sulfoxide concentrations were also examined, due to their use in many of the currently employed therapies to control the spread of HLB. Sublethal heat stress was found to induce the expression of genes related to tryptophan biosynthesis, while repressing the expression of ribosomal proteins. Osmotic stress induces expression of transcriptional regulators involved in expression of extracellular structures, while repressing the biosynthesis of fatty acids and aromatic amino acids. The effects of osmotic stress were further evaluated by quantifying biofilm formation of *L*. *crescens* in presence of increasing sucrose concentrations at different stages of biofilm formation, where sucrose-induced osmotic stress delayed initial cell attachment while enhancing long-term biofilm viability. Our findings revealed that exposure to osmotic stress is a significant contributing factor to the long term survival of *L. crescens* and, possibly, to the pathogenicity of other *Liberibacter* species.

**Supplementary Information:**

The online version contains supplementary material available at 10.1186/s12866-022-02453-w.

## Introduction

Citrus greening has become one of the most devastating citrus diseases in the world. Also known at Huanglongbing (HLB), once infected with this pathogen, there currently is no cure. Widely considered the causative agent, *Liberibacter asiaticus* is transferred from the psyllid vector to the citrus host during feeding on the phloem sap. Due to the inability to sustain an axenic culture, there is limited understanding of this phloem-restricted Alpha-proteobacteria’s physiology [1–3]. Nearly all *Liberibacter* species remain uncultured, however, *L. crescens* BT-1 can be maintained in culture and shares 77.4% nucleotide identity to *L. asiaticus*, with 832 homologous genes. Accordingly, *L. crescens* BT-1 has been thoroughly used as a surrogate strain to provide insights on *L*. *asiaticus* physiology [4, 5].

*L*. *asiaticus* tolerance to fluctuating environmental conditions can be best exemplified by the ability of this pathogen to adapt to the highly variable environmental conditions encountered in the psyllid vector and in the citrus host. Within the psyllid vector, the distribution of *L*. *asiaticus* is nearly systemic, as the bacterium has been identified in many organs and tissues, including the midgut, filter chamber, hindgut, Malpighian tubules, secretory cells of the salivary glands, fat tissues, epidermis, muscle, hemocytes, and neural tissues. Furthermore, *L*. *asiaticus* must tolerate dramatic shifts in osmotic pressure to survive the transfer from the psyllid vector to the phloem of the citrus host. Microscopy and fluorescent in situ hybridization [6–9] has shown that *L*. *asiaticus* forms biofilm-like structures in the psyllid, while agglomerates of cells have been reported to block the phloem sieves in the citrus host [10]. Fluctuations in osmotic strength are very broad within the plant, depending on the tissue (source vs sink). In citrus, sucrose is the major sugar translocated from the leaves to the fruit [11]. It has been reported that the sucrose concentration in citrus phloem are 65.9 mM [12], however, other sugars (such as glucose and fructose) also contribute to the osmotic potential of phloem sap, which can reach sugar concentrations of 103 mM [12]. A broadly found mechanism for bacterial osmoregulation is through the use of channel proteins such as OmpF and OmpC, which are regulated by the transcription factor OmpR [13]. These members are part of a two-component system reciprocally regulated to allow passive diffusion of solutes across the outer membrane [14]. Interestingly, the OmpFCR are absent in both *L*. *crescens* and *L*. *asiaticus* [15], suggesting an alternative mechanism of osmoregulation that may include substantial changes in gene expression that enable the pathogen to survive the extreme environmental changes encountered between hosts. We identified LdtR as a global regulator involved in osmotic stress response in *L*. *asiaticus*, *L. crescens*, and *Sinorhirobium meliloti* [16–19]. LdtR is a member of the MarR-family of transcriptional regulators and was found to control the expression of more than 180 genes in *L*. *asiaticus* [20]. In *Sinorhizobium meliloti*, an insertional mutant of the *ldtR*_*Las*_ homolog, *ldtR*_*Smc*_ (SMc01768), resulted in increased sensitivity to osmotic stress (NaCl and sucrose) and a short-cell phenotype, while increased expression of *ldtR*_*Smc*_ was observed in the wild type when grown under the same osmotic stress conditions [21]. Similarly, Barnett et al. reported reduced swimming motility in a *S*. *meliloti* Δ*ldtR*_*Sme*_ mutant [22]. Using small molecule screening, benzbromarone was identified as a high-affinity ligand that inhibits LdtR activity in vitro and in vivo [17, 21]. Decreased tolerance to osmotic stress was also observed in *S. meliloti* and *L. crescens* in presence of benzbromarone [16].

Unfortunately, therapeutic treatments specific for *L*. *asiaticus* have not been found. Consequently, current treatment for this disease involves a combination of insecticides, antimicrobials, and thermotherapy [23]. The goal of this study was to evaluate global changes in gene expression in response to natural environmental stress or artificially induced stress conditions by agricultural practices. Sucrose was used to induce/mimic long-term osmotic stress conditions that *L*. *asiaticus* may encounter upon entry into the citrus phloem, which contains high concentrations of this organic compound [12]. Physiological responses to long-term heat stress were also examined to further understand the long-term effects of thermotherapy in agriculture management, and the potential development of tolerance and/or survival mechanisms that may become prevalent. Similarly, dimethyl sulfoxide (DMSO) has been used as a carrier for the delivery of new antimicrobial compounds, such as benzbromarone and tolfenamic acid [23]. Gene expression studied under such conditions have the capacity to advance Huanglongbing therapies and uncover the potential of new or combined treatments. Further functional assays, such as biofilm formation, unveiled the antagonistic crosstalk between global regulators of gene expression inhibited by benzbromarone and tolfenamic acid and the osmotic stress.

## Materials and methods

### Growth conditions

*Liberibacter crescens* BT-1 cells were cultured at 26 °C with moderate agitation (180 rpm), in BM7 or modified BM7 (bBM7) media, as previously described [20]. For stress assays, *L. crescens* BT-1 cells were cultured at 26 °C, in BM7 or bBM7 media, supplemented with sucrose (0, 50, 100, 150 or 200 mM), or DMSO (0.01, 0.05, 0.2%). In addition, heat stress was induced by growing cultures at 26 (standard growth condition), 28, 30, and 32 °C. All chemicals were purchased from Sigma-Aldrich (St. Louis, MO, USA).

### Transcriptome analysis

*L. crescens* BT-1 cultures were cultured at 26 °C in standard media, media supplemented with sucrose (100 mM), or media supplemented with DMSO (0.05%). For the heat stress, *L. crescens* cells were grown at 32 °C. When the cultures reached mid-exponential phase (OD_600_ = 0.3), cells were collected by centrifugation at 8,000 rpm, at 4 °C. Total mRNA was extracted with the RiboPure-Bacteria (Life Technologies, Carlsbad, CA, USA) according to the manufacturer’s recommendations. RNA concentration was determined using a NanoDrop One (ThermoFisher Scientific, Waltham, MA USA), and RNA quality was subsequently assessed using the Agilent 2100 Bioanalyzer. rRNA was depleted using the MICROBExpress Bacterial mRNA enrichment kit (Life Technologies) in accordance with the manufacturer’s protocol. Single-end RNA libraries were prepared using the TruSeq Stranded mRNA Library Prep Kit (Illumina, San Diego, CA, USA) followed by sequencing in a HiSeq2500 system. RNA-sequencing was performed at the Genome Sciences Facility, Penn State University, Hershey, PA. The assays were performed in duplicates and approximately 2 million reads were obtained for each library.

Sequencing data was analyzed using a pipeline allocated at the HiPerGator II supercomputer at University of Florida. Briefly, the raw Sanger sequencing data were modified to trim the Illumina sequencing adapter using Cutadapt [24], followed by sequence processing using Sickle [25] to verify the quality of the samples prior to further analyses. Subsequently, all sequences were mapped against the genome of *L. crescens* BT-1 using Bowtie2 [26]. At this point, the remaining rRNA-sequences were depleted in silico. The mapped RNA-sequences were aligned using Samtools [27] and counted using the HTseq tool [28]. The abundance of each transcript from the cells grown in media supplemented with 100 mM sucrose, 0.05% DMSO, or grown at 32 °C was compared against the transcripts from cells grown at 26 °C (control conditions) using the DESeq2 package [29]. Genes with a *p*adj < 0.05 were considered for further analyses. The DE genes identified under each stress condition are shown in the Supplementary Materials (Table S1, S2, S3, S4, S5 and S6).

The functional classification of DE genes was based on the Cluster of Orthologues Genes (COG), as previously described [20]. The percentage of DE genes within each COG was calculated as the number of hits from a given category in the RNA-seq experiments, divided by the total number of genes present within that COG in the genome of *L. crescens* BT-1. Heatmap image was generated from the log2 fold change, using Heatmapper with the default settings. The data discussed in this publication have been deposited in NCBI's Gene Expression Omnibus [30, 31] and are accessible through GEO Series accession number GSE182166 (https://www.ncbi.nlm.nih.gov/geo/query/acc.cgi?acc = GSE182166).

### Biofilm studies

*L. crescens* BT-1 was isolated from papaya and the strain obtained as a gift from Dr. Eric Triplett [4]. *L. crescens* BT-1 cultures were started from a glycerol stock in BM7 media. Cells were grown for 16 h at 26 °C, at 180 rpm. Cells were subcultured in 3 ml of fresh BM7 media at OD_600_ = 0.02. When cultures reached OD_600_ = 0.6, cells were harvested by centrifugation at 4,000 rpm for 10 min at 26 °C. The pellet was washed once by suspending in 3 ml of bBM7 (BM7 media without FBS) and collected by centrifugation as described above. The pellet was subsequently re-suspended at OD_600_ = 0.1 in BM7 or bBM7, supplemented with 0.75 mg/ml of methyl-β-cyclodextrin [32]. Where indicated, BM7 and bBM7 media was supplemented with sucrose (100 mM) or DMSO (0.05%). For subsequent assays, 200 µl of the bacterial cell suspension was used to inoculate 96-well Special Optics Plates (flat bottom, tissue culture treated, black with ultra-thin clear bottom, polystyrene; Corning).

### Confocal Laser Scanning Microscopy (CLSM)

After inoculation, the 96-well Special Optics Plates were sealed with an air permeable AeraSeal film (Excel Scientific), covered with a low-evaporation lid, and placed in the growth chamber with a moisture pack to minimize cross-contamination and evaporation. Plates were incubated at 26 °C, for 2 or 7 days, prior to staining and quantification. Following incubation (2 or 7 dpi), 120 µl of media was removed from each well. Wells were gently washed twice with 200 µl of 0.85% NaCl solution, and samples were subsequently stained using 200 µl of FilmTracer Live/Dead Biofilm Viability Kit (Invitrogen), for 30 min at room temperature, as per the manufacturer’s instructions. After removing the stain, 200 µl of 0.85% NaCl was added for CLSM observation. Biofilm images were collected using a Zeiss LSM800 confocal scanning system with a Plan-Apochromat 20X/0.8 M27 objective. The SYTO-9 and PI fluorophores were exited with an argon laser at 488 nm and 516 nm, respectively, with emission band-pass filters (500 nm and 617 nm, respectively). Parameters for Z-series acquisition were set to the control group (*L. crescens* biofilm in standard BM7 media). Acquisition and processing of images was performed using ZEN Light (Carl Zeiss, Jena, Germany) while quantification of the Z-series images and subsequent biofilms were performed using the COMSTAT2 v2.1 package. Quantification of biomass and thickness was done using data from five Z-stack series, from independent biological replicates (*N* = 5).

### Statistical analyses

The statistical significance of *L. crescens* growth rates were determined using an analysis of variance (ANOVA) and Tukey’s HSD post hoc test. For all experiments, *p* ≤ 0.05 was considered statistically significant, and α = 0.05 was used for Tukey’s HSD tests.

Significance for gene enrichment analyses was conducted using a hypergeometric test [33], where *p* ≤ 0.05 was considered statistically significant.

## Results and discussion

### Identification of stress conditions that alter the growth of *L*. *crescens* BT-1

**Fig. 1:**
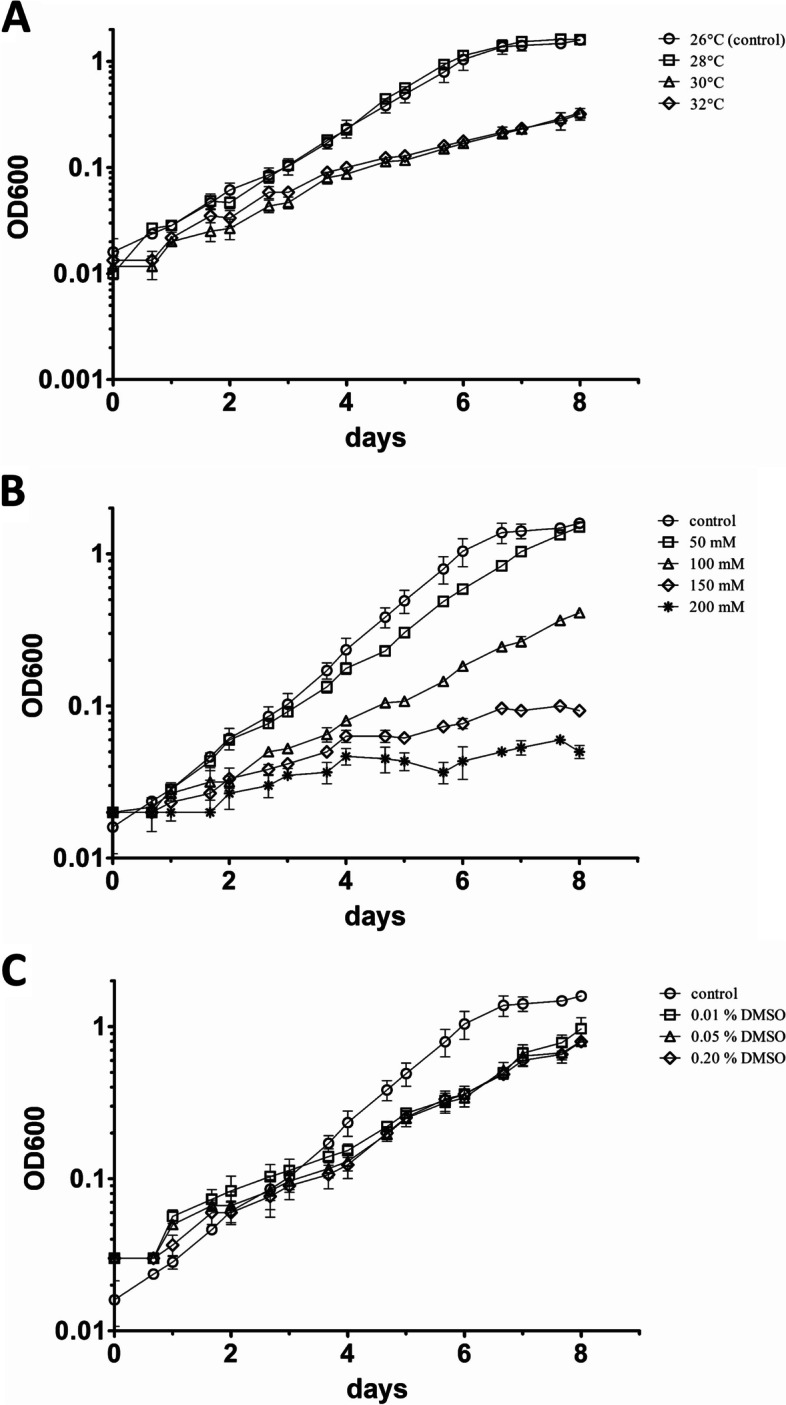
Growth curves of *L. crescens* under the different experimental conditions tested. **A** heat stress (28, 30, and 32 °C); **B** osmotic stress (50, 100, 150, and 200 mM sucrose); and **C** DMSO (0.01, 0.05, and 0.2% DMSO)

The impact of potential environmental or agricultural stressors on *L. crescens* growth was evaluated by determining the growth rate constant (*k*) and the mean generation time (*g*). *L. crescens* was grown under the following conditions: temperature (26, 28, 30 and 32 °C); osmotic stress (50, 100, 150 and 200 mM sucrose); and DMSO (0.01, 0.05 and 0.2%) (Fig. 1). The duplication time of *L. crescens* under standard growth conditions (26 °C) was 25.8 h (Table 1). When the bacterium was grown at 30 or 32 °C, its duplication time was significantly increased by 15.8 h and 22.3 h, respectively. The addition of 50 mM sucrose did not significantly affect the duplication time of *L. crescens* (28.7 h), however, supplementing the media with 100 or 150 mM sucrose significantly increased its doubling time by 14.3 h and 57.8 h, respectively. Very little growth was observed in presence of 200 mM sucrose. The addition of 0.05% and 0.2% DMSO increased the doubling time of *L. crescens* by 8.3 h and 12 h, respectively, however, no significant difference in growth was observed in presence of 0.01% DMSO.

**Table 1:** The effect of heat, sucrose, and/or DMSO in the growth parameters of *L. crescens*

Condition			Growth rate constant, *k*	Mean generation	*p* value
Heat	Sucrose	DMSO	(generation/h)^1^	time, *g* (h)^2^	
26°C^3^	—	—	0.039 ± 0.007	25.8	NS^4^
28 °C	—	—	0.040 ± 0.009	25.1	NS
30 °C	—	—	0.024 ± 0.002	41.6	≤ 0.05
32 °C	—	—	0.021 ± 0.003	48.1	≤ 0.05
—	50 mM	—	0.035 ± 0.003	28.7	NS
—	100 mM	—	0.025 ± 0.001	40.1	≤ 0.05
—	150 mM	—	0.012 ± 0.001	83.6	≤ 0.05
—	200 mM	—	0.007 ± 0.002	142.7	NS
—	—	0.01%	0.034 ± 0.001	29.8	NS
—	—	0.05%	0.029 ± 0.002	34.1	≤ 0.05
—	—	0.20%	0.027 ± 0.003	37.8	≤ 0.05

^1^Growth rate constant (*k*) was calculated from the plot of *log*_*2*_OD_600_ versus time

^2^The mean generation time (*g*) was calculated as 1/*k*

^3^26 °C was considered as control conditions

^4^NS—NS indicates values that did not reach statistical significance

### *L*. *crescens* gene expression profiling under different stressors

**Fig. 2:**
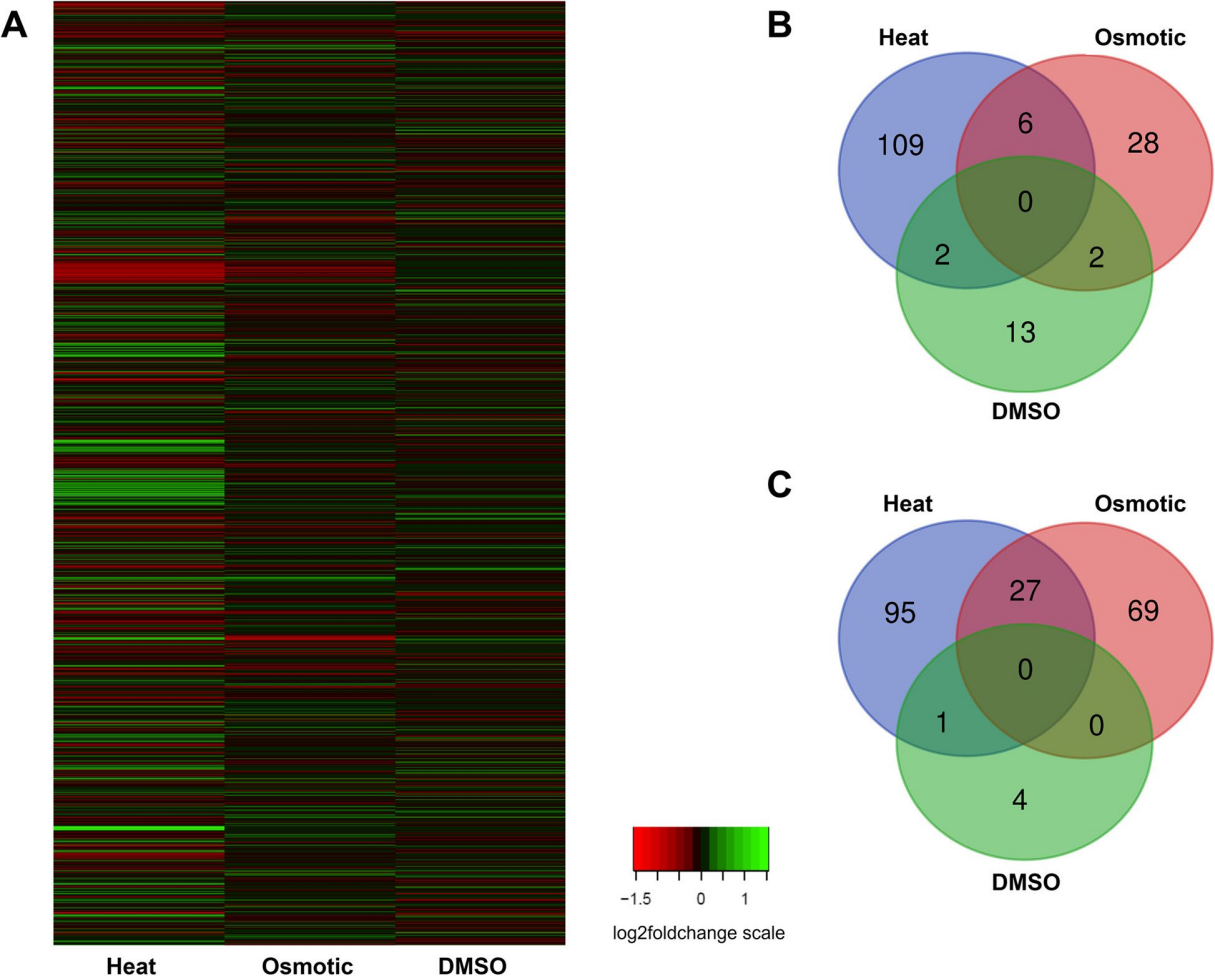
*L. crescens* differentially responds to heat and osmotic stress. **A** Heatmap of differentially expressed genes in response to heat stress, osmotic stress, and DMSO stress. **B**-**C** Venn diagrams of differentially expressed genes that were up-regulated (**B**) or down-regulated (**C**) in response to heat, osmotic, or DMSO stress

Transcriptome profiling was conducted to evaluate the global responses of *L. crescens* to different long-term stress conditions. For every stressor, one condition which significantly modified *k* and *g* parameters (Table 1) was selected for total RNA-seq analysis. *L. crescens* cells were grown to exponential phase under the following three conditions: a) at 32 °C in BM7 (heat stress); b) at 26 °C in BM7 with 100 mM sucrose (osmotic stress); or c) at 26 °C in BM7 with 0.05% DMSO (solvent-vehicle stress). Bacterial cultures grown at 26 °C in BM7 media were used as controls. These stress conditions generated significant (*p*adj ≤ 0.05) changes in gene expression, ranging from threefold induction to twofold repression (Fig. 2A, Table S7). The range of fold change obtained is in line with our previous reports involving chemical inactivation of transcriptional regulators in *L. crescens* [20, 32]. As previously suggested, a contributing factor to these small changes in gene expression may be the high duplication time of *L. crescens,* which further leads to low transcriptional activity.

To compare the variance of the normalized read counts among all the tested conditions, a principal component analysis (PCA) was conducted, where the transcriptomic data from each biological replicate clustered together (Fig. S1). Interestingly, the read counts from the heat stress clustered away from the other stress conditions, explaining 43% of the variance in PC1. DMSO did not induce drastic changes in gene expression and was observed to cluster in close proximity to the controls. These results suggest that *L. crescens* responds differently to heat and osmotic stress, while the addition of 0.05% DMSO has marginal effects on gene expression.

The number of differentially expressed (DE) genes, as defined by genes with a *p*adj value ≤ 0.05, was analyzed for each of the different stress conditions (Table S7). Heat stress caused the largest number of DE genes (240), with 117 up-regulated and 123 down-regulated genes (Table S1 and S2, respectively). Osmotic stress resulted in 132 DE genes, with 36 up-regulated and 96 down-regulated genes (Table S3 and S4, respectively). The stress generated by DMSO resulted in the smallest number of DE genes (22 genes), with 17 up-regulated and 5 down-regulated genes (Table S5 and S6, respectively).

Venn diagrams were generated to analyze the DE genes that are common between the three conditions tested, where six up-regulated genes were shared between the osmotic stress group and heat stress treatment groups, and only two up-regulated genes were shared among the heat and DMSO groups, and the osmotic and DMSO groups (Fig. 2B). 93% of the DE genes (109 genes) identified under heat stress were unique to this treatment group, while 78% of the DE genes (28 genes) were unique for the osmotic treatment group, and 77% (13 genes) were unique for the DMSO group. No up-regulated genes were common among all three stress conditions (Fig. 2B). 28% of the down-regulated genes (27 genes) in the heat stress group were shared among the osmotic treatment groups (Fig. 2C), while only 1 down-regulated gene (*B488_RS03150*), annotated as cold shock protein CspG, was shared between the heat stress and DMSO treatment groups. No down-regulated genes were common among all stress conditions (Fig. 2C). Altogether, no specific pathway was enriched between treatments, indicating that the responses to each of the stress conditions tested is specific.

**Fig. 3:**
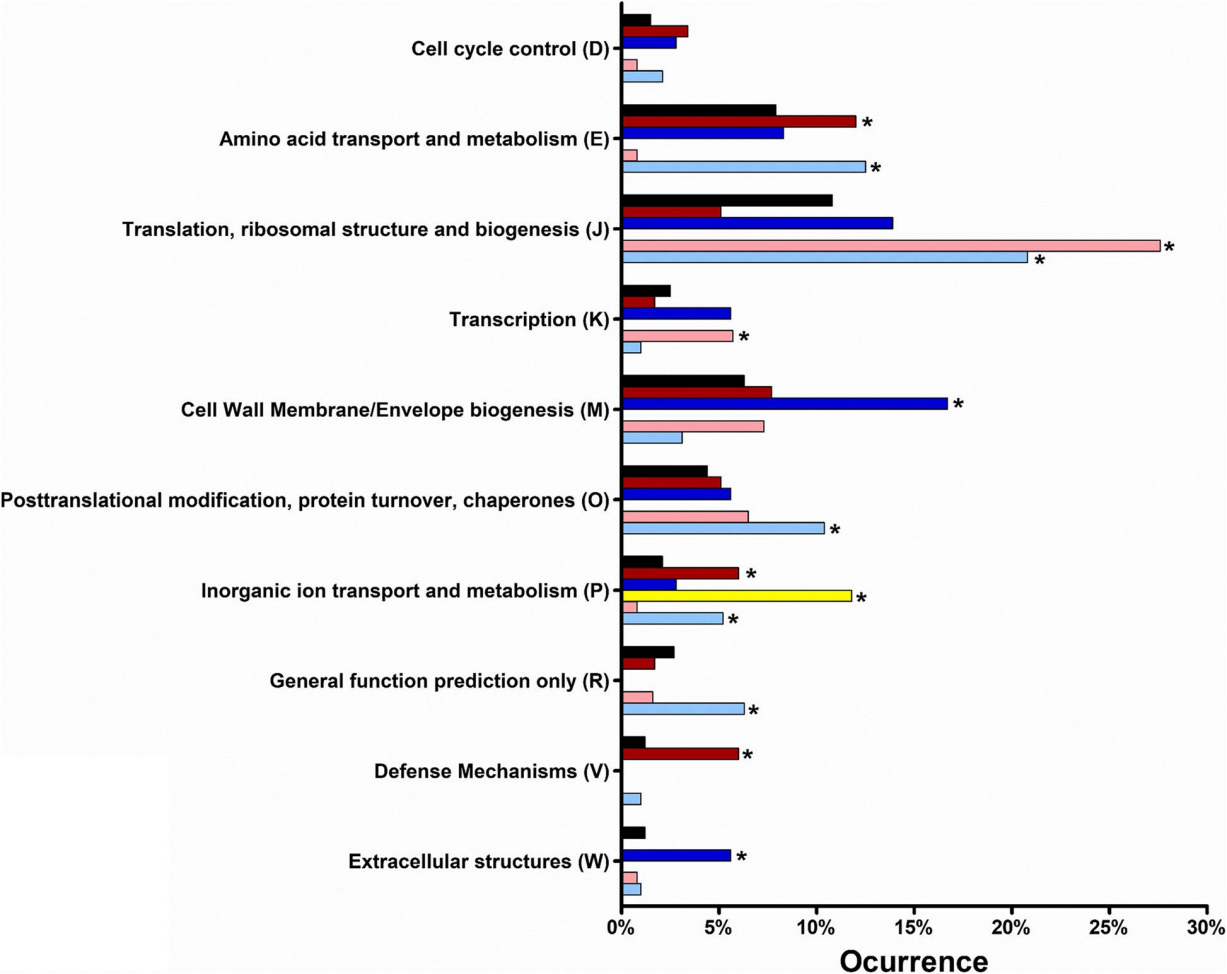
Functional classification of differentially expressed genes in *L. crescens* in response to heat stress, osmotic stress, and DMSO stress. The classification, based on the Cluster of Orthologous genes (COG), in each COG relative to the whole genome (black bars). Within each COG, up-regulated genes are shown for heat stress (dark red), osmotic stress (dark blue), and DMSO (yellow). Within each COG, down-regulated genes are shown for heat stress (light red) and osmotic stress (light blue). ^*^COG groups that were significantly (*p* ≤ 0.05) enriched in the hypergeometric test

To evaluate the functional role of the genes identified, all of the protein coding genes were retrieved from the *L. crescens* BT-1 genome and organized according to the Cluster of Orthologous Genes (COG) categories [34]; the identified DE genes under each stress condition were then organized according to the COG categories. To determine which COG categories were enriched or diminished among the DE genes, a hypergeometric test [33] was conducted (Table S8). COG categories which were significantly enriched by at least one stress type are summarized in Fig. 3 and denoted with an asterisk.

### Heat stress induces tryptophan biosynthesis and represses translation machinery

Long-term heat stress induced up-regulation of a significant (*p* ≤ 0.05) number of genes from the following COG categories: amino acid transport (E), nucleotide transport (F), translation (J), inorganic ion transport (P), and defense mechanisms (V) (Fig. 3, Table S1 and S8). The categories for cell cycle control (D) and cell wall membrane biogenesis (M) showed many up-regulated genes despite not being statistically significant (*p* = 0.07 and *p* = 0.11, respectively). Subsequently, we searched within these categories for putative transcriptional units that were up-regulated in response to heat. We identified an operon involved in the biosynthesis of L-tryptophan (encoded by *trpA-trpB-trpF-trpD-trpG-trpE*, *B488_RS06360*–*B488_RS06385*, Table S1). The regulation of the tryptophan operon (*trp*), mediated by the Trp repressor and transcriptional attenuation, has been widely studied in bacteria [35]. *Bacillus subtilis* harbors a temperature-sensitive tryptophanyl-tRNA synthetase where it was observed that at high temperatures, when the bacterium growth is reduced but not suppressed, the *trp* operon was induced [36]. This process is likely mediated by the reduced availability of the *trp* attenuator protein TRAP [37, 38]. However, *L. crescens* does not encode for any homologs of the Trp repressor or TRAP, thus the mechanism by which heat induces the expression of *trp* genes in *L. crescens* has yet to be identified.

The only methionine (D-methionine) transport system annotated and identified in the genome of *L. crescens* (*metN-metI-metQ*) was also recognized in this subset of DE genes (Table S1). In *E. coli* this locus can import both L- and D- methionine [39] whose uptake is usually regulated by the internal methionine pool size [40]. In *Vibrio choler*a D-methionine is incorporated in cell wall muropeptide [41], a process associated with the strengthening of the cell wall. D-methionine is also one of the most effective D-amino acids involved in mechanisms of biofilm disassembly in both *B. subtilis* and *Staphylococcus epidermis* [42, 43]. Increments in the D-methionine pool, and perhaps the L- enantiomer too, may be involved in the strengthening of the cell wall in *L. crescens* as a response to heat stress. The zinc/manganese ABC transporter systems *znu*_2_ and *znu*_3_ [19] were up-regulated under heat stress. In our previous transcriptomic analysis, where LdtR was chemically inhibited, we observed up-regulation of the *znu*_3_ system, but down-regulation of the *znu*_2_ system [20], which suggest these genes possess more than one regulatory mechanism.

Heat stress caused the significant downregulation of a number of genes from the translation, ribosomal structure and biogenesis (J), and transcription (K) groups (Fig. 3, Table S2 and S8). The following putative operons were identified to be down-regulated in response to heat stress: the chromosome partitioning proteins (*parA* and *parB, B488_RS00020* and *B488_RS00025*, respectively); the lipid A biosynthetic pathway (*yaeT-lpxD-fabZ-B488_RS00250, B488_RS00235–B488_RS00250*); twenty-six 30S and 50S ribosomal proteins; the RNA polymerase beta and beta-prime subunits (*B488_RS04035* and *rpoB, B488_RS04040*) and sigma factor (*rpoD, B488_RS06505*); as well as an operon comprising the DNA binding protein and proteases *hubP*-*lon*-*clpX* (*B488_RS04370*–*B488_RS04380*). Among single genes that were down-regulated were the Xre- and MucR-family transcriptional regulators (*B488_RS00800* and *B488_RS00180*, respectively) and an iron-sulfur cluster assembly machinery protein (*iscA, B488_RS04630*).

Taken together, these results suggest that *L. crescens* responds to heat stress by inducing the synthesis of tryptophan and the uptake of D-methionine. It has been reported that membrane proteins have a significantly higher tryptophan content when compared to cytoplasmic proteins [44]. It is possible that the increment in the tryptophan and methionine pool observed during heat stress is related to the biosynthesis of membrane proteins. The transcriptional repression of the components of the RNA polymerase complex and many of the ribosomal proteins involved in translation observed under heat stress explains the notable increased duplication time of the bacteria at increasing temperatures (Table 1).

Heat stress through the use of thermotherapy, was found to be most effective in reducing *L*. *asiaticus* DNA titers when conducted at 55 °C for 90 to 120 s, or at 60 °C for 30 s [45]. However, the ability to reach such high temperatures in large field applications remains challenging. Our findings involving *L*. *crescens* gene expression reveal an untapped potential to combine treatments involving heat stress (thermotherapy) with inhibitors of key physiological pathways such as tryptophan biosynthesis and methionine import. This could alleviate physical damage to citrus tissue, allow for a faster recovery time, and allow for a potential reduction in heat or heat application time when the treatments are used in parallel.

### Osmotic stress induces expression of transcriptional regulators involved in expression of extracellular structures, while repressing the biosynthesis of fatty acids and aromatic amino acids

The hypergeometric test revealed that the extracellular structures (W) and cell wall membrane biogenesis (M) categories were significantly enriched among up-regulated genes (8 genes) in response to long-term osmotic stress (Fig. 3, Table S3 and S8). Genes involved in pilus assembly and adherence, *pilA* and *tadB* (*B488_RS06285* and *B488_RS06235*, respectively), were among the genes induced, as well as cell wall membrane/envelope biogenesis proteins *yidC* (*B488_RS06430*) and *cmeC* (*B488_RS04790*), and a septum formation protein *B488_RS06730*. While the category transcription (K) did not have statistical significance (*p* = 0.169), several transcriptional and translational regulators, including *fur/zur*, *visN*, *dksA*, and *pleD,* were significantly up-regulated (1.27, 1.27, 1.25, and 1.33-fold, respectively, *p*adj < 0.05) (Table S3). Interestingly, some of these proteins have been reported to regulate some processes of the enriched COG categories, and were also found to control the expression of stress responses, cell cycle, and biogenesis of extracellular elements [32]. While *L*. *asiaticus* does not encode for homologs of *fur*/*zur*, *dksA*, or *pleD*, it is possible that other regulators sustain these functions. A previous report on the relative expression of *L*. *asiaticus* genes in the citrus host versus the psyllid identified 11 upregulated transcription factors, with the two highest being *ldtR* and *phoU* [46]. In agreement with this report, our results in *L*. *crescens* revealed that *ldtR* and the leucine-responsive regulatory protein *lrp* were both up-regulated under osmotic stress conditions, however, these differences were not statistically significant (*p*adj = 0.47 and *p*adj = 0.37, respectively). We have previously shown that inactivation of LdtR in *L. crescens* results in reduced osmotic tolerance. The apparent discrepancy between our previous results and the presented transcriptomics data may be explained by fluctuations in the temporal expression of LdtR.

*B488_RS06210* was originally annotated as a transcriptional regulator of the *fur* (ferric uptake regulator) family, however, a detailed sequence alignment identified the zinc uptake repressor protein (Zur) as the best hit. This family of regulators controls zinc homeostasis by activating or repressing zinc transporter systems based on metal availability [47]. In *E. coli* K-12, Zur regulates the expression of the *znuABC* operon, the *L31* and *L36* ribosomal proteins, and the periplasmic zinc binding protein *zinT* [48]. While osmotic stress caused the induction of ribosomal proteins and repression of the *znu*_2_ system (Table S3 and S4), no homologs of ZinT were found in the genome of *L. crescens*. While there is little information available regarding the role of Zur regulation during osmotic stress, dysregulation of the zinc uptake system in *Neisseria meningitidis* severely impacted its ability to form biofilms [49]; metal mobilization and its effects on biofilm have also been seen in *Pseudomonas aeruginosa*, where dysregulation of iron mobilization impaired biofilm formation, regardless of environmental availability [50].

We observed that the expression of *visN* is significantly upregulated during osmotic stress (1.27-fold, *p*adj < 0.05). Similar results were reported for the VisN homolog in *L*. *asiaticus* when transitioning from the psyllid to the citrus host [46]. It was also reported that the VisNR system negatively regulates the expression of the *flp3* pilus in *L*. *asiaticus* [51], and their involvement was proposed in the initial attachment and colonization of the bacterium within the host. In agreement with these observations, we recently reported that in *L. crescens*, the global regulator PrbP binds to the promoter region of *visN* and up-regulates its expression under biofilm forming conditions [32]. PrbP was also found to induce the expression of *mucR*, consequently repressing the expression of *rem*, an activator of motility genes [32]. While VisN has been reported to form a heterodimer with VisR to regulate gene expression in other bacterial systems [51, 52], recent evidence suggests that in *L*. *crescens* VisN and VisR are not expressed as a single transcript [32]. In agreement with these findings, here we found that under osmotic stress, the expression of *visR* (*B488_RS00900*) was not affected.

The expression of *visN* (*B488_RS00615*) is significantly upregulated during osmotic stress (1.25- fold, *p*adj < 0.05). DksA is a key regulatory protein in *E. coli* associated with dehydration tolerance and protection against dehydration-associated stress [53]. The stringent response regulator DksA regulates gene expression via binding to the RNA polymerase secondary channel [54] and plays a crucial role in the induction of the general stress response regulator RpoS [55]. While *L*. *asiaticus* and *L. crescens* do not have a gene encoding for *rpoS*, expression of the commonly associated (p)ppGpp synthetase *relA* was not significantly affected in *L. crescens* under osmotic stress, suggesting DksA plays a role during the osmotic stress response, independent of the small signaling molecule ppGpp.

PleD is a two-component response regulator encoded by *B488_RS00385*. The expression of this gene was significantly upregulated during osmotic stress (1.33-fold, *p*adj < 0.05). Its homolog in *Caulobacter crescentus* coordinates polar differentiation [56]. It contains a GGDEF domain, which is responsible for the generation of cyclic-di-guanosine monophosphate (c-di-GMP). This signaling molecule has been extensively associated to the regulation of processes such as biofilm formation and motility [57, 58], and the localization of PleD to the cell pole is dependent on its phosphorylation state as well as its diguanylate cyclase activity. *L. crescens* encodes four GGDEF domain proteins, but only *pleD* was found to change expression during osmotic stress. Interestingly, osmotic-induced stress did not affect expression levels of the known putative PleD kinases, *pleC* and *divJ* (*B488_RS06115* and *B488_RS04425*, respectively), suggesting other kinases may be responsible for the phosphorylation of PleD under these stress conditions.

Among the up-regulated operons observed in response to osmotic stress, we found members of a putative unit encoding two high-affinity iron transporters, a cupredoxin, and a ferredoxin (*B488_RS04450*–*B488_RS04465*, Table S3) where *B488*_*RS04465* was upregulated but did not reach statistical significance (*p* = 0.065). Interestingly, in *B*. *subtilis* and *Jeotgalibacillus malaysiensis*, the expression of genes related to iron uptake was also observed when bacteria were subjected to osmotic stress [59, 60]. This effect could in part explain the reversed growth defect observed in salt-stressed *B. subtilis*, while it was speculated that iron may be crucial in *J. malaysiensis* for the redox center of enzymes involved in the respiratory chain.

Osmotic stress decreased the expression of genes classified in several COG categories (Fig. 3, Table S4 and S8), including amino acid transport (E), translation, ribosomal structure and biogenesis (J), post-translational modification, protein turnover, chaperones (O), inorganic ion transport (P), and general function (R). The category carbohydrate transport (G), was enriched among down-regulated genes, however, it did not reach statistical significance (*p* = 0.06). Other down-regulated genes included the cell cycle transcriptional regulator *ctrA* (*B488_RS05010*), iron uptake regulator *fur* (*B488_RS00775*), nitrogen-related response regulator *ntrC* (*B488_RS02845*), and polar-differentiation response regulator *divK* (*B488_RS00380*). CtrA is a global response regulator that has been extensively studied for its role in the bacterial cell cycle, as well as in the biogenesis of pili and flagellar structures [61–64]. In *S. meliloti*, gene expression profiling of *ctrA*, at different stages of symbiosis, revealed the expression of *ctrA* transcripts were down-regulated during bacteroid differentiation within the nodule [65, 66], where the bacteria may be exposed to higher concentrations of sucrose and undergoing cellular differentiation. In *C. crescentus*, activity of the cell cycle regulator CtrA is controlled by the response regulator DivK, as well as the protease adapter CpdR [67, 68]. While DivK modulates the kinase and phosphatase activities of sensor histidine kinases, CpdR functions as a modulator of proteolysis. During our osmotic stress experiments, *divK* and *cdpR* (encoded by *B488_RS00380* and *B488_RS05135*, respectively) were both down-regulated, however, only *divK* reached statistical significance (*p* = 0.04).

The expression of *fur* (*B488_RS00775*) was significantly downregulated during osmotic stress (1.27 fold, *p*adj < 0.05). It belongs to the family of transcriptional regulators of ferric uptake and negatively controls the expression of iron uptake proteins and siderophores [69]. The decrease in *fur* mRNA expression can be linked to the up-regulation of the high-affinity iron transporters, cupredoxin and ferredoxin, described above. The gene *B488_RS02845* encodes a sigma-54 dependent transcriptional activator (*atoC*), that was one of the most abundant transcripts in our analysis, and was found to be down-regulated in response to osmotic stress (Table S4). In *E. coli*, AtoC is involved in the catabolism of short-chain fatty acids and expression of flagellar genes [70, 71]. While the role of AtoC during osmotic stress remains unclear, during the analysis of down-regulated operons we identified a fatty acid biosynthesis gene cluster (*B488_RS04895*–*B488_RS04915*) that may be linked to the down-regulation of *atoC*.

Among the down-regulated operons was a zinc/manganese ABC transporter system *znu*_2_ (*B488_RS04920*–*B488_RS04935*, Table S4), which is likely mediated by the up-regulation of Zur. The chaperones *groES* (*B488_RS01265*) and *groEL* (*B488_RS01270*) were also found to be down-regulated by osmotic stress. These results are in contrast to the expression reported for these genes in *L. asiaticus,* where the GroEL homolog was found to be up-regulated in the phloem of the citrus host when compared to its expression in the psyllid vector [46].

It was also observed that several TCA-cycle enzymes, including dihydrolipoamide dehydrogenase, 2-oxoglutarate dehydrogenase, two succinyl-CoA synthetases and malate dehydrogenase (*B488_RS03575*, *ldpC*; *B488_RS03580*, *sucA*; *B488_RS03590*, *sucD*; *B488_RS03595, sucC*; and *B488_RS03600*, *mdh*, respectively) were also down-regulated. Of note, some of the genes from the last putative operons although not having a *p*adj value smaller than 0.05, displayed fold changes below 1 under this stress condition. Nine ribosomal genes were also found to be differentially expressed in response to osmotic stress, all of which were down-regulated (Table S4).

Two genes involved in cell division, *ftsZ* (*B488_RS02910*) and *ftsA* (*B488_RS02905*), were also significantly down-regulated in response to sucrose stress. FtsZ is the bacterial homolog of eukaryotic tubulin and is an essential cell division protein that forms the cytokinetic ring, while FtsA is a peripheral membrane protein that localizes nearby and anchors FtsZ to the membrane [72]. Several post-translational modification enzymes were also down-regulated in response to sucrose, including serine protease *htrA* (*B488_RS06080*), peptidyl-prolyl cis–trans isomerase C and D (*prsA* and *surA*, *B488_RS01645* and *B488_RS01515*, respectively), and proteases S41 (*B488_RS01675*) and ClpB (*B488_RS01410*).

Several genes from the shikimate pathway were also down-regulated in response to osmotic stress, including shikimate kinase (*aroK*, *B488_RS05515*), shikimate dehydrogenase (*aroE*, *B488_RS06725*) and 3-phosphoshikimate 1-carboxyvinyltransferase (*B488_RS00545*). Since these genes play a role in the biosynthesis of folate and aromatic amino acids, the down-regulation of this group of genes may be due to reduced duplication rates/turnover and decreased protein synthesis under these growth conditions. In *V. cholerae*, subpopulations of growth-arrested and persister bacteria were observed during osmotic shock with limited nutrient availability [73], while in *Streptomyces*, hyperosmotic shock triggered arrest of growth, loss of cell turgor, and hyper condensation of the chromosomes [74]. Taken together, our findings suggest that *L. crescens* may respond similarly to sucrose-induced osmotic stress, by inducing several transcriptional regulators involved in the formation of extracellular structures and by repressing cell division and biosynthesis of fatty acids and aromatic amino acids.

### DMSO as a vehicle control has a minor impact host gene expression

Dimethyl sulfoxide is unique solvent that is commonly used in agricultural crop management programs to prepare fertilizers, pesticides, herbicides and bactericidal compounds [23, 75–79]. Recent studies have also examined benzbromarone and tolfenamic acid formulations containing 1% DMSO, that were administered to citrus trees by trunk injection and foliar spray, to help combat citrus greening disease [23]. In this study, the effect of DMSO was examined in *L. crescens*, where only 22 genes were differential expressed during growth in presence of 0.05% DMSO, which represents less than 2% of the entire *L. crescens* genome (Table S5, S6 and S7). Inorganic ion transport (P) was the only category significantly enriched during long-term DMSO stress (Fig. 3, Table S8); *pstS* (*B488_RS06150*) and *ftn* (*B488_RS06215*) were the only DE genes in this category. PstS is responsible for binding phosphate in the periplasm and acting as a phosphate ABC transporter, while Ftn is an iron-storage protein. No putative operons were found among the DE genes (up- or down-regulated). These results suggest that DMSO does not have a significant impact on gene expression in *L. crescens,* at the concentration tested.

### Osmotic stress delays initial cell attachment while enhancing long-term biofilm viability

**Fig. 4:**
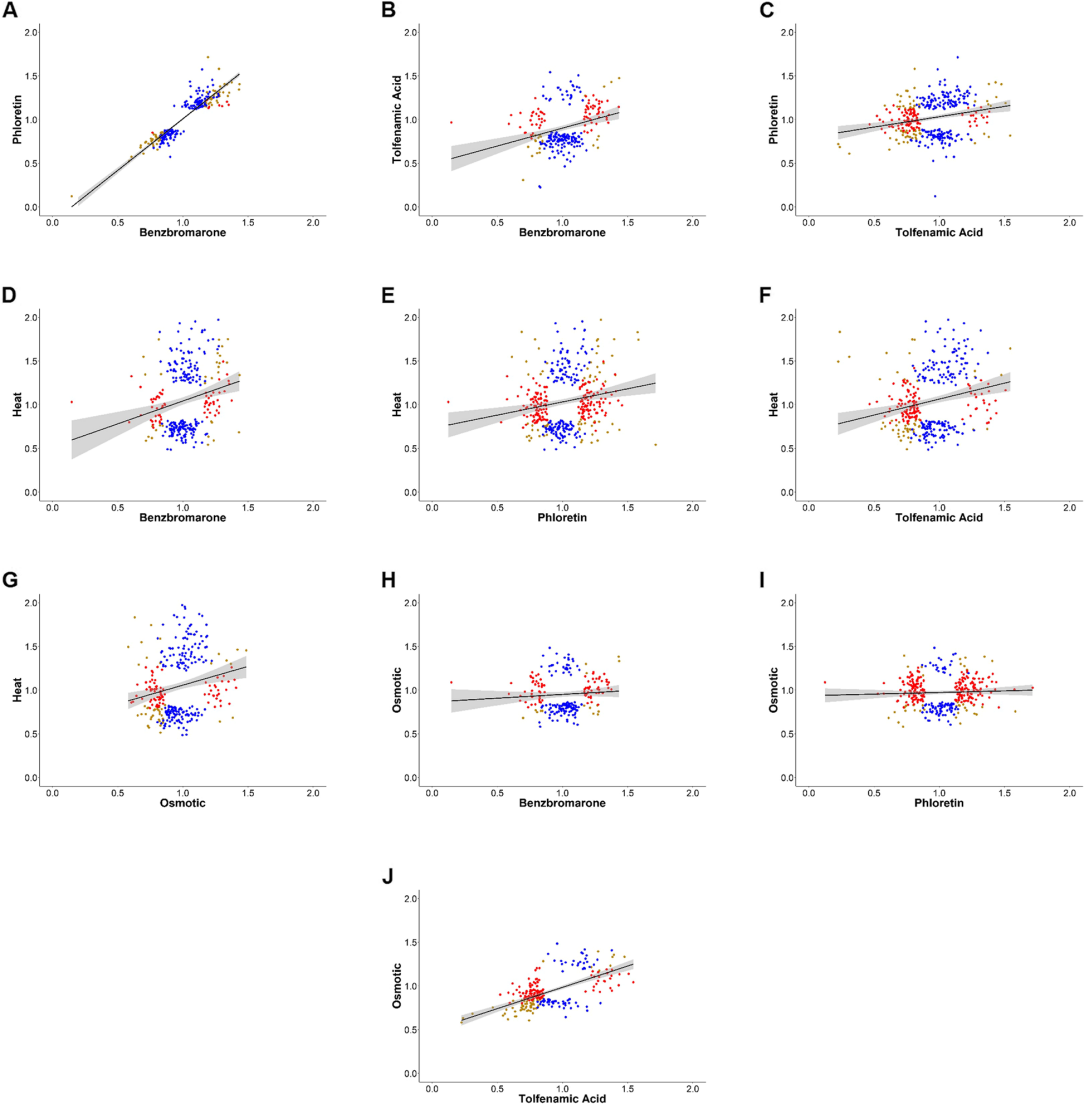
Correlation test between significant (*p* ≤ 0.05) differentially expressed (DE) genes in *L. crescens* following growth under stress conditions (heat, osmotic, or DMSO) and antimicrobial treatments (benzbromarone, phloretin, or tolfenamic acid). DE genes corresponding to the stressors indicated on the X-axis are shown in red; DE genes corresponding to the stressors indicated on the Y-axis are shown in blue. DE genes that were identified in both conditions are shown in gold

While evaluating several environmental stressors in this study, we identified several DE transcriptional regulators involved in the formation of extracellular structures (Fig. 4J), thus we hypothesized that osmotic stress could potentially alter biofilm formation. To determine the impact of long-term osmotic stress on biofilm formation, *L. crescens* was grown to exponential phase as previously described [32]. Following growth in BM7 media, cells were transferred to BM7 or bBM7 (biofilm forming media), in absence or presence (100 mM) of sucrose, and incubated for 12 h. A summary of the experimental layout design is depicted in Fig. S2. The cultures were subsequently divided into three subgroups; cells grown in standard media without sucrose supplementation, cells grown in media supplemented with 100 mM sucrose after the 12 h incubation period (–/ +), and cells grown in media supplemented with 100 mM sucrose during and after the 12 h incubation period (+ / +). At two- and seven-days post-incubation, the biofilm was quantified by live/dead staining in combination with confocal laser scanning microscopy (CLSM). Confocal Z-stack images were obtained for the quantification and reconstruction of 2D and 3D visualization of the biofilm.

**Fig. 5:**
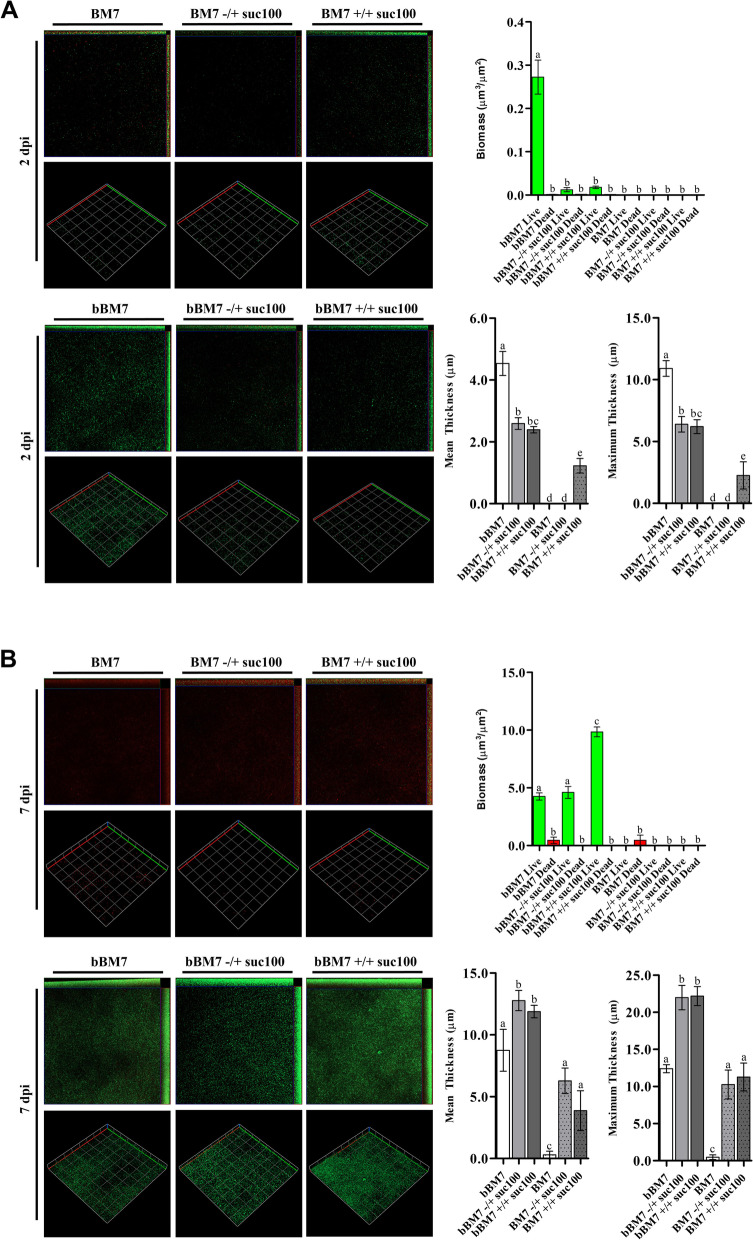
Osmotic stress reduces cell attachment and biofilm formation in *L*. *crescens*, while increasing long-term survival. CLSM observations and quantification of *L. crescens* biofilms were performed in absence or presence (100 mM) of sucrose. Cell viability was evaluated using Filmtracer Live/Dead stain at (**A**) 2 and (**B**) 7 days post-incubation (dpi). Live and dead cells are indicated by green and red bars, respectively. BM7, non-biofilm forming media. bBM7, biofilm forming media. Growth conditions are indicated for cells grown in BM7 or bBM7 media without sucrose supplementation (–) or with 100 mM sucrose supplementation ( +) as follows: cells grown in standard media before and after the 12 h incubation; cells exposed to sucrose-supplemented media after the 12 h incubation (–/ +); and cells incubated with sucrose-supplemented media during and after the 12 h incubation (+ / +). Cells incubated in standard BM7 or bBM7 were used as the controls. Representative 2D and 3D views were rendered from Z-series of at least three independent biological replicates. Quantification of CLSM observations were performed using ImageJ and Comstat2 v2.1 package. Statistical significance was determined from at least three independent biological replicates as described in the materials and methods. Statistical significance (*p* ≤ 0.05) is indicated by different letters above each bar

At two days post-incubation (dpi), biofilm biomass was observed in samples suspended in standard bBM7 media and in samples incubated with bBM7 supplemented with 100 mM sucrose (–/ + and + / +), where cells inoculated in standard bBM7 were found to have significantly (*p* ≤ 0.05) higher amounts biomass (0.27 ± 0.10 µm^3^ µm^−2^), mean thickness (4.48 ± 0.99 µm), and maximum thickness (11.28 ± 1.32 µm) when compared to cells incubated with bBM7 supplemented with sucrose (Fig. 5A). No significant difference was observed between bBM7 groups treated with sucrose (–/ + and + / +). No significant difference in biomass was observed in any of the samples grown in BM7, however, a significant increase in mean thickness (1.22 ± 0.52 µm) and maximum thickness (2.25 ± 2.45 µm) was observed in BM7 cultures that were supplemented with 100 mM sucrose throughout the experiment (+ / +), indicating the sucrose-enriched BM7 media increased sedimentation of bacterial cells, but they were unable to transition into a biofilm state (Fig. 5A).

At 7 dpi, the mean thickness, maximum thickness, and biomass of cells incubated in bBM7 supplemented with sucrose (+ / +) was significantly (*p* ≤ 0.05) higher than the bBM7 control, while bBM7 (–/ +) only showed a significant increase in the mean and maximum thickness. Live biomass was significantly (*p* ≤ 0.05) increased in bBM7 supplemented with sucrose (+ / +) when compared to bBM7 control (9.84 ± 0.85 and 4.25 ± 0.67 µm^3^ µm^−2^, respectively) (Fig. 5B). In samples incubated with sucrose-supplemented bBM7 after the 12 h incubation period (–/ +), the live biomass (4.60 ± 1.06 µm^3^ µm^−2^) was similar to the bBM7 control. In the bBM7 group, the mean thickness (4.74 ± 3.79 µm) and maximum thickness (12.40 ± 1.22 µm) were significantly (*p* ≤ 0.05) lower than the bBM7 (–/ +) and (+ / +) groups (12.77 ± 1.63 and 11.88 ± 1.00 µm, respectively) (Fig. 5B). Similar to samples measured at 2 dpi, there was no quantifiable biomass in any of the BM7 groups at 7 dpi (Fig. 5B), however, in the BM7 (–/ +) and (+ / +) groups, a mean thickness of (7.76 ± 0.62 and 3.87 ± 2.27 µm) and maximum thickness of (14.57 ± 3.32 and 11.28 ± 2.65 µm) was observed (Fig. 5B). Taken together, these results suggest that osmotic stress delayed the attachment of cells, but enhanced long-term biomass survival, as seen after 7 dpi.

### Gene profile observed during sucrose stress is similar to the chemical inactivation of PrbP

PrbP is a transcriptional regulatory protein that is involved in the regulation of diverse cellular processes in *L. asiaticus* and *L. crescens*, and was recently found to play a critical role in *L. crescens* during transition from a motile to sessile state [32]. The addition of tolfenamic acid prevented biofilm formation in *L. crescens*. Members of the PrbP and LdtR regulons were identified by changes in gene expression in presence of specific ligands in *L. crescens*. Distinct transcript profiles were observed using sub-lethal concentrations of benzbromarone (Benz) and phloretin (Phlor), ligands of LdtR [20], and tolfenamic acid (Tolf), a ligand of PrbP [32]. To examine potential overlaps between the DE genes observed in response to chemical-inactivation treatments and those observed in this study (in response to long-term heat and osmotic stress), linear regression analysis was conducted among all of the DE genes. Analysis of the DE genes identified following treatment with inhibitors of LdtR (Benz and Phlor), showed a positive correlation (*R*^*2*^ = 0.85, Fig. 4A and Table S9), confirming the high similarity in gene expression response. In contrast, comparison of the DE genes identified while using an inhibitor of PrbP (Tolf) with those identified in presence of LdtR inhibitors (Benz or Phlor) showed no correlation (Tolf vs Benz, *R*^*2*^ = 0.09; Tolf vs Phlor, *R*^*2*^ = 0.06, Fig. 4B, C and Table S9). These results suggest that under the conditions tested, the response to chemical inactivation of LdtR and PrbP affect different subsets of genes, despite similar COGs being enriched by each treatment [32]. No correlation was observed between the DE genes identified following heat stress when compared to Phlor, Tolf, or osmotic stress (Heat vs Benz, *R*^*2*^ = 0.05; Heat vs Phlor, *R*^*2*^ = 0.03; Heat vs Tolf, *R*^*2*^ = 0.05; Heat vs Osmotic, *R*^*2*^ = 0.04; Fig. 4D-G and Table S9). Surprisingly, DE genes in response to osmotic stress and chemical inactivation of LdtR show no correlation (Benz vs Osmotic, *R*^*2*^ < 0.01; Phlor vs Osmotic, *R*^*2*^ < 0.01; Fig. 4H, I and Table S9) despite sharing several enriched COG categories. We have previously reported that LdtR inactivation resulted is growth defects in both *S. meliloti* and *L. crescens* [21].

Interestingly, the inactivation of PrbP by tolfenamic acid showed positive correlation with the transcriptional response generated by osmotic stress (Tolf vs Osmotic, *R*^*2*^ = 0.37, Fig. 4J). Accounting for this positive correlation are genes from COG categories paramount in cell proliferation, survival, adaptation, and biofilm formation, highlighting the role of PrbP in cell survival as previously reported [32]. We recently reported that the inactivation of PrbP leads to an absence of biofilm formation while the exposure to osmotic stress decreased initial cell attachment. Therefore, the positive correlation seen between PrbP and osmotic stress at the transcriptional level explains the contrasting results obtained in biofilm formation.

## Conclusion

*L. asiaticus* has adapted to tolerate drastic changes in osmolarity during its life cycle within the citrus phloem and during transmission between the psyllid vector and citrus host [46]. While the mechanism which enables tolerance to these extreme changes in environmental conditions is yet to be fully elucidated, herein we show that long-term osmotic stress is a significant driving factor behind changes in gene expression that facilitates cell adhesion, biofilm formation, and increased long-term viability in *L. crescens*. While *L*. *asiaticus* does not maintain a canonical system for osmoregulation, the changes seen in *L*. *crescens* provide a steppingstone in the understanding of such complex system. Proceeding reports describe the response to chemical inactivation of LdtR and PrbP in *L. crescens*, where some COG categories were enriched in both treatments, however, each was found to affect a different subset of genes. One explanation for this occurrence may be the temporal difference in which the observed DE genes exert their cardinal regulatory roles. This rationale may also explain why the chemical inactivation of LdtR showed little correlation with the genes identified during osmotic stress. Interestingly, chemical inactivation of PrbP revealed expression patterns similar to osmotic stress in cells during exponential phase, which is likely the cause for the delayed biofilm formation observed in cells exposed to elevated sucrose concentrations. Osmotic pressure is a key factor in the induction of biofilm formation in other species, including *V*. *cholerae*, *S*. *epidermidis*, and *B*. *subtilis* [80–82], where pleiotropic changes in expression profiles and cell attachment were observed in response to variations in osmotic stress [83]. Taken together, we hypothesize that exposure to increased osmotic pressure is a significant contributing factor in the pathogenicity of *Liberibacter* species and may contribute to colonization within the citrus host, while simultaneously increasing resistance to plant defense mechanisms and antimicrobial therapies.

## Supplementary Information

**Additional file 1: Table S1.**

**Additional file 2: Table S2. **

**Additional file 3: Table S3. **

**Additional file 4: Table S4.**

**Additional file 5: Table S5. **

**Additional file 6: Table S6.**

**Additional file 7: Table S7. **

**Additional file 8: Figure S1.**

**Additional file 9: Table S8.**

**Additional file 10: Figure S2.**

**Additional file 11: Table S9.**

## Data Availability

The data discussed in this publication have been deposited in NCBI's Gene Expression Omnibus [30, 31] and are accessible through GEO Series accession number GSE182166 (https://www.ncbi.nlm.nih.gov/geo/query/acc.cgi?acc=GSE182166).
