# Supplementary material for: Osmotic stress induces long-term biofilm survival in Liberibacter crescens
Source: BMC Microbiol. 2022 Feb 11;22:52. doi: 10.1186/s12866-022-02453-w (PMC8832773; doi:10.1186/s12866-022-02453-w)
Supplement: Supplementary file 1 — Additional file 1: Table S1. [file 12866_2022_2453_MOESM1_ESM.docx]

**Table S1.** Up-regulated genes in *L. crescens* under heat stress.

| **Locus tag** | **Fold Change** | ***p*adj** | **Annotation** | **COG** |
| --- | --- | --- | --- | --- |
| B488_RS00095 | 1.49 | 0.05 | homoserine kinase | Signal transduction mechanisms |
| B488_RS00360 | 1.28 | 0.05 | peptide chain release factor 2 | Translation, ribosomal structure and biogenesis |
| B488_RS00430 | 1.38 | 0.01 | hypothetical protein | noCOG |
| B488_RS00480 | 1.29 | 0.01 | branched-chain amino acid transport system substrate-binding protein | Amino acid transport and metabolism |
| B488_RS00590 | 1.23 | 0.02 | hypothetical protein | General function prediction only |
| B488_RS00630 | 1.28 | 0.04 | Uncharacterized membrane protein | Function unknown |
| B488_RS00640 | 1.83 | 4.48E-14 | ATP-binding cassette, subfamily B | Defense Mechanisms |
| B488_RS00645 | 1.31 | 0.03 | mannosyltransferase | Cell Wall Membrane/Envelope biogenesis |
| B488_RS00685 | 1.38 | 0.01 | pyridoxine 5-phosphate synthase | Coenzyme transport and metabolism |
| B488_RS00690 | 1.27 | 0.04 | small subunit ribosomal protein S21 | Translation, ribosomal structure and biogenesis |
| B488_RS00695 | 1.29 | 0.01 | Tetratricopeptide repeat-containing protein | noCOG |
| B488_RS00980 | 1.62 | 0.03 | hypothetical protein | noCOG |
| B488_RS01115 | 1.75 | 3.38E-06 | tRNA(Arg) A34 adenosine deaminase TadA | Translation, ribosomal structure and biogenesis |
| B488_RS01140 | 1.93 | 2.50E-06 | hypothetical protein | noCOG |
| B488_RS01275 | 2.47 | 3.11E-06 | hypothetical protein | noCOG |
| B488_RS01320 | 1.32 | 0.04 | NADH-quinone oxidoreductase subunit H | Energy production and conversion |
| B488_RS01415 | 1.48 | 4.15E-03 | Peptidase family M23 | Cell Wall Membrane/Envelope biogenesis |
| B488_RS01475 | 2.26 | 8.86E-05 | hypothetical protein | noCOG |
| B488_RS01480 | 1.39 | 0.03 | Major Facilitator Superfamily protein | Amino acid transport and metabolism |
| B488_RS01630 | 1.41 | 0.01 | peptide/nickel transport system permease protein | Amino acid transport and metabolism |
| B488_RS01680 | 1.28 | 0.05 | hypothetical protein | Carbohydrate transport and metabolism |
| B488_RS01690 | 1.35 | 0.03 | choline-sulfatase | Inorganic ion transport and metabolism |
| B488_RS01725 | 1.27 | 0.03 | TolA protein | Intracellular trafficking, secretion, and vesicular transport |
| B488_RS01840 | 1.30 | 0.03 | ATP-binding cassette, subfamily C | Intracellular trafficking, secretion, and vesicular transport |
| B488_RS01930 | 1.53 | 5.75E-05 | glutamine synthetase | Amino acid transport and metabolism |
| B488_RS02405 | 1.31 | 0.01 | hypothetical protein | noCOG |
| B488_RS02640 | 1.48 | 0.05 | dUTP pyrophosphatase | Defense Mechanisms |
| B488_RS02645 | 1.47 | 0.01 | magnesium chelatase family protein | Posttranslational modification, protein turnover, chaperones |
| B488_RS02720 | 1.39 | 0.01 | 2-octaprenyl-6-methoxyphenol hydroxylase | Coenzyme transport and metabolism |
| B488_RS02735 | 1.43 | 0.02 | hypothetical protein | noCOG |
| B488_RS02740 | 1.95 | 2.11E-07 | HlyD family secretion protein | Cell Wall Membrane/Envelope biogenesis |
| B488_RS02745 | 1.75 | 2.04E-03 | putative ABC transport system ATP-binding protein | Cell Wall Membrane/Envelope biogenesis |
| B488_RS02750 | 1.45 | 0.01 | putative ABC transport system permease protein | Defense Mechanisms |
| B488_RS02790 | 1.40 | 0.03 | hypothetical protein | noCOG |
| B488_RS02860 | 1.35 | 0.03 | N-methyl-L-tryptophan oxidase | Amino acid transport and metabolism |
| B488_RS03150 | 1.25 | 0.01 | cold shock protein (beta-ribbon, CspA family) | Transcription |
| B488_RS03155 | 1.38 | 0.01 | hypothetical protein | noCOG |
| B488_RS03195 | 1.31 | 0.02 | DNA helicase-2 / ATP-dependent DNA helicase PcrA | Replication, recombination and repair |
| B488_RS03395 | 2.36 | 2.53E-07 | type I restriction enzyme M protein | Defense Mechanisms |
| B488_RS03405 | 1.86 | 0.01 | type I restriction enzyme, R subunit | Defense Mechanisms |
| B488_RS03410 | 1.61 | 0.02 | Transposase | Mobilome: prophages, transposons |
| B488_RS03435 | 1.41 | 0.01 | UDP-N-acetylglucosamine-N-acetylmuramylpentapeptide N-acetylglucosamine transferase | Cell Wall Membrane/Envelope biogenesis |
| B488_RS03445 | 1.62 | 8.91E-04 | UDP-N-acetylmuramoylalanine--D-glutamate ligase | Cell Wall Membrane/Envelope biogenesis |
| B488_RS03450 | 1.41 | 1.89E-03 | Phospho-N-acetylmuramoyl-pentapeptide-transferase | Cell Wall Membrane/Envelope biogenesis |
| B488_RS03460 | 1.50 | 1.31E-03 | UDP-N-acetylmuramoylalanyl-D-glutamate--2,6-diaminopimelate ligase | Cell Wall Membrane/Envelope biogenesis |
| B488_RS03465 | 1.32 | 0.01 | cell division protein FtsI (penicillin-binding protein 3) | Cell cycle control |
| B488_RS03475 | 1.32 | 0.05 | 16S rRNA (cytosine1402-N4)-methyltransferase | Translation, ribosomal structure and biogenesis |
| B488_RS03620 | 1.42 | 4.54E-04 | succinate dehydrogenase subunit A | Energy production and conversion |
| B488_RS03630 | 1.64 | 2.33E-06 | succinate dehydrogenase subunit C | Energy production and conversion |
| B488_RS03640 | 1.59 | 4.71E-03 | glycerol-3-phosphate dehydrogenase (NAD(P)+) | Energy production and conversion |
| B488_RS03655 | 2.26 | 2.58E-10 | uroporphyrinogen-III synthase | Coenzyme transport and metabolism |
| B488_RS03665 | 1.35 | 0.01 | HemY protein | Function unknown |
| B488_RS03690 | 1.48 | 3.30E-03 | cytochrome b561 | Energy production and conversion |
| B488_RS03730 | 1.60 | 0.04 | hypothetical protein | noCOG |
| B488_RS03750 | 1.61 | 0.03 | hypothetical protein | noCOG |
| B488_RS03765 | 1.67 | 0.01 | putative portal protein | noCOG |
| B488_RS03770 | 1.67 | 0.01 | terminase large (ATPase) subunit and inactivated derivatives | Mobilome: prophages, transposons |
| B488_RS03795 | 2.03 | 0.00E+00 | replicative DNA helicase | Replication, recombination and repair |
| B488_RS03805 | 1.97 | 4.84E-07 | hypothetical protein | noCOG |
| B488_RS03810 | 1.74 | 2.87E-03 | zinc/manganese transport system permease protein | Inorganic ion transport and metabolism |
| B488_RS03815 | 1.74 | 3.38E-06 | zinc/manganese transport system substrate-binding protein - Znu3 | Inorganic ion transport and metabolism |
| B488_RS03820 | 1.43 | 7.17E-04 | hypothetical protein | noCOG |
| B488_RS03880 | 1.59 | 0.01 | hypothetical protein | noCOG |
| B488_RS03890 | 1.35 | 0.04 | exodeoxyribonuclease-5 | noCOG |
| B488_RS03925 | 1.87 | 1.27E-08 | Protein of unknown function (DUF1217) | noCOG |
| B488_RS04030 | 1.50 | 1.60E-04 | ATP-binding cassette, subfamily B, multidrug efflux pump | Defense Mechanisms |
| B488_RS04170 | 1.31 | 0.02 | Fe-S cluster assembly protein SufD | Posttranslational modification, protein turnover, chaperones |
| B488_RS04210 | 1.41 | 0.02 | Pimeloyl-ACP methyl ester carboxylesterase | Coenzyme transport and metabolism |
| B488_RS04410 | 1.44 | 0.01 | ABC-type uncharacterized transport system, substrate-binding protein | General function prediction only |
| B488_RS04415 | 1.34 | 0.04 | hypothetical protein | noCOG |
| B488_RS04450 | 1.46 | 4.37E-04 | hypothetical protein | Cell Wall Membrane/Envelope biogenesis |
| B488_RS04455 | 1.46 | 0.01 | Cupredoxin-like domain-containing protein | noCOG |
| B488_RS04465 | 1.34 | 0.03 | 4Fe-4S binding domain-containing protein | Energy production and conversion |
| B488_RS04480 | 1.64 | 2.79E-03 | hypothetical protein | noCOG |
| B488_RS04530 | 1.53 | 0.01 | flagellar hook-associated protein 1 FlgK | Cell Motility |
| B488_RS04575 | 1.32 | 0.02 | putative acyl-CoA dehydrogenase | Lipid transport and metabolism |
| B488_RS04680 | 1.36 | 0.01 | alcohol dehydrogenase | Energy production and conversion |
| B488_RS04685 | 1.31 | 0.04 | 3-oxoacyl-[acyl-carrier-protein] synthase II | Lipid transport and metabolism |
| B488_RS04825 | 1.53 | 0.02 | hypothetical protein | noCOG |
| B488_RS04895 | 1.25 | 0.02 | UPF0755 protein | Cell cycle control |
| B488_RS04920 | 1.83 | 1.94E-09 | manganese/iron transport system substrate-binding protein - Znu2 | Inorganic ion transport and metabolism |
| B488_RS04925 | 1.49 | 3.21E-06 | manganese/iron transport system ATP-binding protein | Inorganic ion transport and metabolism |
| B488_RS04930 | 1.55 | 2.09E-06 | manganese/iron transport system permease protein | Inorganic ion transport and metabolism |
| B488_RS05120 | 1.48 | 4.83E-04 | D-methionine transport system ATP-binding protein - MetN | Amino acid transport and metabolism |
| B488_RS05130 | 1.59 | 6.07E-05 | D-methionine transport system substrate-binding protein - MetQ | Inorganic ion transport and metabolism |
| B488_RS05425 | 1.64 | 5.45E-07 | ATP-binding cassette, subfamily B | Posttranslational modification, protein turnover, chaperones |
| B488_RS05480 | 1.34 | 0.02 | Methyltransferase domain-containing protein | noCOG |
| B488_RS05535 | 1.85 | 4.84E-06 | hypothetical protein | noCOG |
| B488_RS05555 | 1.51 | 1.35E-03 | hypothetical protein | noCOG |
| B488_RS05640 | 1.31 | 0.03 | 23S rRNA (guanosine2251-2'-O)-methyltransferase | Translation, ribosomal structure and biogenesis |
| B488_RS05650 | 1.28 | 0.03 | translocation and assembly module TamB | Intracellular trafficking, secretion, and vesicular transport |
| B488_RS05660 | 1.59 | 8.69E-08 | Glycine zipper | noCOG |
| B488_RS05675 | 1.26 | 0.02 | DNA segregation ATPase FtsK/SpoIIIE, S-DNA-T family | Cell cycle control |
| B488_RS05690 | 1.40 | 0.03 | 3',5'-cyclic AMP phosphodiesterase CpdA | Signal transduction mechanisms |
| B488_RS05695 | 1.35 | 0.01 | 23S rRNA pseudouridine1911/1915/1917 synthase | Translation, ribosomal structure and biogenesis |
| B488_RS05855 | 1.29 | 0.03 | molecular chaperone DnaK | Posttranslational modification, protein turnover, chaperones |
| B488_RS05900 | 1.70 | 6.98E-07 | hypothetical protein | noCOG |
| B488_RS05975 | 1.56 | 6.96E-05 | peptide-methionine (R)-S-oxide reductase | Posttranslational modification, protein turnover, chaperones |
| B488_RS06080 | 1.27 | 0.01 | serine protease Do | Posttranslational modification, protein turnover, chaperones |
| B488_RS06115 | 1.25 | 0.05 | two-component system, cell cycle sensor histidine kinase PleC | Signal transduction mechanisms |
| B488_RS06360 | 1.75 | 0.01 | tryptophan synthase, alpha chain - TrpA | Amino acid transport and metabolism |
| B488_RS06365 | 2.49 | 3.38E-06 | tryptophan synthase beta chain - TrpB | Amino acid transport and metabolism |
| B488_RS06370 | 2.30 | 2.00E-05 | phosphoribosylanthranilate isomerase /indole-3-glycerol phosphate synthase - TrpF | Amino acid transport and metabolism |
| B488_RS06375 | 2.13 | 1.35E-04 | anthranilate phosphoribosyltransferase - TrpD | Amino acid transport and metabolism |
| B488_RS06380 | 2.30 | 3.33E-05 | anthranilate synthase component 2 - TrpG | Amino acid transport and metabolism |
| B488_RS06385 | 1.75 | 0.01 | anthranilate synthase, component I - TrpE | Amino acid transport and metabolism |
| B488_RS06465 | 1.48 | 7.67E-04 | 3-oxoacyl-[acyl-carrier-protein] synthase-3 | Lipid transport and metabolism |
| B488_RS06470 | 1.37 | 3.31E-04 | peroxiredoxin, Ohr subfamily | Defense Mechanisms |
| B488_RS06495 | 1.63 | 0.03 | hypothetical protein | noCOG |
| B488_RS06540 | 1.49 | 2.10E-03 | Tetratricopeptide repeat-containing protein | noCOG |
| B488_RS06545 | 1.42 | 0.02 | integral membrane efflux protein | noCOG |
| B488_RS06760 | 2.91 | 2.94E-32 | BA14K-like protein | noCOG |
| B488_RS06800 | 1.32 | 0.03 | hypothetical protein | noCOG |
| B488_RS06805 | 1.64 | 1.12E-05 | hypothetical protein | noCOG |
| B488_RS06810 | 1.62 | 9.53E-07 | Hemolysin-type calcium-binding repeat-containing protein | Secondary metabolites biosynthesis, transport and catabolism |
| B488_RS06815 | 1.84 | 3.80E-03 | Hemolysin-type calcium-binding region | Secondary metabolites biosynthesis, transport and catabolism |
| B488_RS06870 | 1.48 | 0.04 | hypothetical protein | noCOG |
