# Supplementary material for: Osmotic stress induces long-term biofilm survival in Liberibacter crescens
Source: BMC Microbiol. 2022 Feb 11;22:52. doi: 10.1186/s12866-022-02453-w (PMC8832773; doi:10.1186/s12866-022-02453-w)
Supplement: Supplementary file 2 — Additional file 2: Table S2. [file 12866_2022_2453_MOESM2_ESM.docx]

**Table S2.** Down-regulated genes in *L. crescens* under heat stress.

| **Locus tag** | **Fold Change** | ***p*adj** | **Annotation** | **COG** |
| --- | --- | --- | --- | --- |
| B488_RS00020 | 0.56 | 1.70E-07 | chromosome partitioning protein - ParA | Cell Motility |
| B488_RS00025 | 0.66 | 1.58E-03 | chromosome partitioning protein, ParB family - ParB | Cell cycle control |
| B488_RS00045 | 0.75 | 0.03 | hypothetical protein | General function prediction only |
| B488_RS00055 | 0.81 | 0.04 | hypothetical protein | Function unknown |
| B488_RS00080 | 0.57 | 3.94E-03 | DNA-binding response regulator, OmpR family, contains REC and winged-helix (wHTH) domain | Signal transduction mechanisms |
| B488_RS00180 | 0.76 | 0.01 | transcriptional regulator, MucR family - MucR | Transcription |
| B488_RS00215 | 0.76 | 0.01 | ribosome recycling factor | Translation, ribosomal structure and biogenesis |
| B488_RS00220 | 0.81 | 0.05 | undecaprenyl diphosphate synthase | Lipid transport and metabolism |
| B488_RS00235 | 0.83 | 0.04 | Beta-barrel assembly machine subunit BamA - YaeT | Cell Wall Membrane/Envelope biogenesis |
| B488_RS00240 | 0.79 | 0.03 | UDP-3-O-[3-hydroxymyristoyl] glucosamine N-acyltransferase - LpxD | Cell Wall Membrane/Envelope biogenesis |
| B488_RS00245 | 0.71 | 0.01 | 3-hydroxyacyl-[acyl-carrier-protein] dehydratase - FabZ | Lipid transport and metabolism |
| B488_RS00250 | 0.73 | 2.75E-03 | acyl-[acyl-carrier-protein]--UDP-N-acetylglucosamine O-acyltransferase | Cell Wall Membrane/Envelope biogenesis |
| B488_RS00380 | 0.75 | 0.02 | two-component system, cell cycle response regulator DivK | Signal transduction mechanisms |
| B488_RS00510 | 0.75 | 0.05 | sulfur carrier protein | Coenzyme transport and metabolism |
| B488_RS00570 | 0.72 | 0.03 | DNA polymerase-3 subunit beta | Replication, recombination and repair |
| B488_RS00600 | 0.74 | 3.34E-04 | aconitase | Energy production and conversion |
| B488_RS00800 | 0.80 | 0.03 | Transcriptional regulator, contains XRE-family HTH domain - Xre | Transcription |
| B488_RS00850 | 0.74 | 1.21E-03 | large subunit ribosomal protein L25 | Translation, ribosomal structure and biogenesis |
| B488_RS00860 | 0.66 | 1.08E-03 | ferredoxin | Function unknown |
| B488_RS00875 | 0.66 | 5.10E-05 | PAS domain S-box-containing protein | Transcription |
| B488_RS00925 | 0.67 | 0.02 | chemotaxis protein MotA | Cell Motility |
| B488_RS01365 | 0.74 | 0.01 | prolyl-tRNA synthetase | Translation, ribosomal structure and biogenesis |
| B488_RS01495 | 0.78 | 0.05 | Pyridoxamine 5'-phosphate oxidase | Coenzyme transport and metabolism |
| B488_RS01665 | 0.65 | 2.77E-04 | ribosome-associated protein | Translation, ribosomal structure and biogenesis |
| B488_RS01765 | 0.72 | 0.03 | hypothetical protein (DUF2155) | noCOG |
| B488_RS01780 | 0.76 | 0.02 | ATP-dependent Clp protease, protease subunit | Posttranslational modification, protein turnover, chaperones |
| B488_RS01870 | 0.66 | 2.50E-06 | glutamine synthetase | Amino acid transport and metabolism |
| B488_RS01885 | 0.71 | 1.81E-03 | single-strand DNA-binding protein | Replication, recombination and repair |
| B488_RS01900 | 0.74 | 2.04E-03 | Glycosyltransferase, GT2 family | Carbohydrate transport and metabolism |
| B488_RS01955 | 0.72 | 0.01 | L-aspartate 1-decarboxylase | Coenzyme transport and metabolism |
| B488_RS01995 | 0.61 | 2.37E-06 | SSU ribosomal protein S10P | Translation, ribosomal structure and biogenesis |
| B488_RS02000 | 0.69 | 4.76E-05 | large subunit ribosomal protein L3 | Translation, ribosomal structure and biogenesis |
| B488_RS02005 | 0.62 | 3.11E-06 | large subunit ribosomal protein L4 | Translation, ribosomal structure and biogenesis |
| B488_RS02010 | 0.59 | 1.78E-06 | large subunit ribosomal protein L23 | Translation, ribosomal structure and biogenesis |
| B488_RS02015 | 0.73 | 4.00E-04 | large subunit ribosomal protein L2 | Translation, ribosomal structure and biogenesis |
| B488_RS02020 | 0.71 | 0.01 | small subunit ribosomal protein S19 | Translation, ribosomal structure and biogenesis |
| B488_RS02025 | 0.71 | 1.20E-03 | large subunit ribosomal protein L22 | Translation, ribosomal structure and biogenesis |
| B488_RS02030 | 0.69 | 7.49E-05 | small subunit ribosomal protein S3 | Translation, ribosomal structure and biogenesis |
| B488_RS02035 | 0.59 | 1.30E-09 | large subunit ribosomal protein L16 | Translation, ribosomal structure and biogenesis |
| B488_RS02040 | 0.64 | 1.19E-04 | large subunit ribosomal protein L29 | Translation, ribosomal structure and biogenesis |
| B488_RS02045 | 0.59 | 4.84E-07 | small subunit ribosomal protein S17 | Translation, ribosomal structure and biogenesis |
| B488_RS02050 | 0.68 | 4.33E-04 | large subunit ribosomal protein L14 | Translation, ribosomal structure and biogenesis |
| B488_RS02055 | 0.75 | 0.01 | large subunit ribosomal protein L24 | Translation, ribosomal structure and biogenesis |
| B488_RS02060 | 0.63 | 1.51E-05 | large subunit ribosomal protein L5 | Translation, ribosomal structure and biogenesis |
| B488_RS02065 | 0.59 | 2.11E-07 | small subunit ribosomal protein S14 | Translation, ribosomal structure and biogenesis |
| B488_RS02070 | 0.63 | 2.65E-07 | small subunit ribosomal protein S8 | Translation, ribosomal structure and biogenesis |
| B488_RS02075 | 0.72 | 1.17E-03 | large subunit ribosomal protein L6 | Translation, ribosomal structure and biogenesis |
| B488_RS02080 | 0.60 | 4.38E-07 | large subunit ribosomal protein L18 | Translation, ribosomal structure and biogenesis |
| B488_RS02085 | 0.72 | 2.10E-03 | small subunit ribosomal protein S5 | Translation, ribosomal structure and biogenesis |
| B488_RS02095 | 0.57 | 3.38E-09 | large subunit ribosomal protein L15 | Translation, ribosomal structure and biogenesis |
| B488_RS02100 | 0.76 | 0.01 | protein translocase subunit secY/sec61 alpha | Intracellular trafficking, secretion, and vesicular transport |
| B488_RS02105 | 0.75 | 0.01 | Adenylate kinase | Nucleotide transport and metabolism |
| B488_RS02110 | 0.79 | 0.03 | small subunit ribosomal protein S13 | Translation, ribosomal structure and biogenesis |
| B488_RS02125 | 0.65 | 1.37E-06 | large subunit ribosomal protein L17 | Translation, ribosomal structure and biogenesis |
| B488_RS02225 | 0.78 | 0.04 | Uncharacterized conserved protein, DUF2336 family | Function unknown |
| B488_RS02545 | 0.81 | 0.02 | small subunit ribosomal protein S4 | Translation, ribosomal structure and biogenesis |
| B488_RS02560 | 0.64 | 6.71E-05 | monothiol glutaredoxin | Posttranslational modification, protein turnover, chaperones |
| B488_RS02565 | 0.76 | 0.02 | Stress-induced morphogen (activity unknown) | Signal transduction mechanisms |
| B488_RS02590 | 0.66 | 1.02E-04 | phosphoribosylaminoimidazole-succinocarboxamide synthase | Nucleotide transport and metabolism |
| B488_RS06830 | 0.69 | 4.68E-05 | outer membrane immunogenic protein | Cell Wall Membrane/Envelope biogenesis |
| B488_RS02865 | 0.64 | 0.01 | hypothetical protein (DUF2312) | noCOG |
| B488_RS02870 | 0.65 | 2.21E-03 | modulator of drug activity B | General function prediction only |
| B488_RS02915 | 0.54 | 8.46E-14 | UDP-3-O-[3-hydroxymyristoyl] N-acetylglucosamine deacetylase | Cell Wall Membrane/Envelope biogenesis |
| B488_RS02920 | 0.71 | 4.02E-04 | Beta-barrel assembly machine subunit BamD | Cell Wall Membrane/Envelope biogenesis |
| B488_RS02925 | 0.78 | 0.03 | DNA repair protein RecN (Recombination protein N) | Replication, recombination and repair |
| B488_RS03065 | 0.69 | 0.01 | conserved hypothetical protein | noCOG |
| B488_RS03320 | 0.78 | 0.05 | glucosamine--fructose-6-phosphate aminotransferase (isomerizing) | Cell Wall Membrane/Envelope biogenesis |
| B488_RS03565 | 0.67 | 4.26E-05 | hypothetical protein | Function unknown |
| B488_RS03595 | 0.81 | 0.05 | succinyl-CoA synthetase (ADP-forming) beta subunit | Energy production and conversion |
| B488_RS03675 | 0.62 | 3.53E-04 | 6-pyruvoyltetrahydropterin/6-carboxytetrahydropterin synthase | Coenzyme transport and metabolism |
| B488_RS03980 | 0.72 | 0.03 | hypothetical protein | noCOG |
| B488_RS03990 | 0.49 | 1.04E-17 | hypothetical protein | noCOG |
| B488_RS04035 | 0.81 | 0.02 | DNA-directed RNA polymerase subunit beta' | Transcription |
| B488_RS04040 | 0.80 | 0.01 | DNA-directed RNA polymerase subunit beta - RpoB | Transcription |
| B488_RS04045 | 0.73 | 5.86E-04 | large subunit ribosomal protein L7/L12 | Translation, ribosomal structure and biogenesis |
| B488_RS04055 | 0.80 | 0.02 | LSU ribosomal protein L1P | Translation, ribosomal structure and biogenesis |
| B488_RS04100 | 0.67 | 2.43E-05 | D-3-phosphoglycerate dehydrogenase | Coenzyme transport and metabolism |
| B488_RS04300 | 0.76 | 0.03 | hypothetical protein | noCOG |
| B488_RS04370 | 0.71 | 1.94E-03 | DNA-binding protein HU-beta - HubP | Replication, recombination and repair |
| B488_RS04375 | 0.77 | 2.04E-03 | ATP-dependent Lon protease - Lon | Posttranslational modification, protein turnover, chaperones |
| B488_RS04380 | 0.78 | 0.01 | ATP-dependent Clp protease ATP-binding subunit ClpX - ClpX | Posttranslational modification, protein turnover, chaperones |
| B488_RS04525 | 0.65 | 0.03 | flagellar hook-associated protein 3 FlgL | Cell Motility |
| B488_RS04570 | 0.78 | 0.03 | flagellin | Cell Motility |
| B488_RS04600 | 0.78 | 0.02 | transaldolase | Carbohydrate transport and metabolism |
| B488_RS04630 | 0.49 | 2.82E-07 | Iron-sulfur cluster assembly accessory protein - IscA | Posttranslational modification, protein turnover, chaperones |
| B488_RS04635 | 0.64 | 1.51E-04 | exodeoxyribonuclease-3 | Replication, recombination and repair |
| B488_RS04655 | 0.71 | 0.03 | protein-L-isoaspartate(D-aspartate) O-methyltransferase | Posttranslational modification, protein turnover, chaperones |
| B488_RS04700 | 0.78 | 0.03 | acyl carrier protein | Lipid transport and metabolism |
| B488_RS04705 | 0.76 | 4.28E-03 | aspartyl/glutamyl-tRNA(Asn/Gln) amidotransferase subunit B | Translation, ribosomal structure and biogenesis |
| B488_RS04715 | 0.73 | 0.01 | aspartyl/glutamyl-tRNA(Asn/Gln) amidotransferase subunit C | Translation, ribosomal structure and biogenesis |
| B488_RS04820 | 0.63 | 3.15E-03 | hypothetical protein | noCOG |
| B488_RS04855 | 0.62 | 1.88E-03 | dTDP-4-dehydrorhamnose 3,5-epimerase | Cell Wall Membrane/Envelope biogenesis |
| B488_RS04905 | 0.77 | 0.01 | acyl carrier protein | Lipid transport and metabolism |
| B488_RS04950 | 0.72 | 3.23E-03 | inorganic pyrophosphatase | Energy production and conversion |
| B488_RS04975 | 0.79 | 0.01 | trigger factor | Posttranslational modification, protein turnover, chaperones |
| B488_RS05010 | 0.80 | 0.03 | cell cycle transcriptional regulator CtrA - CtrA | Signal transduction mechanisms |
| B488_RS05160 | 0.51 | 1.76E-18 | Porin subfamily protein | noCOG |
| B488_RS05185 | 0.72 | 1.21E-03 | large subunit ribosomal protein L20 | Translation, ribosomal structure and biogenesis |
| B488_RS05190 | 0.72 | 5.76E-04 | large subunit ribosomal protein L35 | Translation, ribosomal structure and biogenesis |
| B488_RS05195 | 0.78 | 0.01 | translation initiation factor IF-3 | Translation, ribosomal structure and biogenesis |
| B488_RS05225 | 0.78 | 0.05 | phosphoribosylformylglycinamidine cyclo-ligase | Nucleotide transport and metabolism |
| B488_RS05275 | 0.77 | 0.04 | NusB antitermination factor | Transcription |
| B488_RS05315 | 0.80 | 0.04 | porphobilinogen synthase | Coenzyme transport and metabolism |
| B488_RS05325 | 0.78 | 0.01 | citrate synthase | Energy production and conversion |
| B488_RS05355 | 0.76 | 0.01 | pyruvate dehydrogenase E1 component alpha subunit | Energy production and conversion |
| B488_RS05360 | 0.82 | 0.03 | pyruvate dehydrogenase E1 component beta subunit | Energy production and conversion |
| B488_RS05390 | 0.64 | 1.95E-05 | sec-independent protein translocase protein TatA | Intracellular trafficking, secretion, and vesicular transport |
| B488_RS05540 | 0.72 | 0.01 | hypothetical protein | noCOG |
| B488_RS05600 | 0.81 | 0.01 | outer membrane immunogenic protein | Cell Wall Membrane/Envelope biogenesis |
| B488_RS05730 | 0.69 | 1.95E-04 | F-type H+-transporting ATPase subunit gamma | Energy production and conversion |
| B488_RS05785 | 0.80 | 0.04 | demethylmenaquinone methyltransferase / 2-methoxy-6-polyprenyl-1,4-benzoquinol methylase | Coenzyme transport and metabolism |
| B488_RS05915 | 0.73 | 0.01 | regulator of CtrA degradation | Function unknown |
| B488_RS06150 | 0.75 | 2.10E-03 | phosphate transport system substrate-binding protein | Inorganic ion transport and metabolism |
| B488_RS06285 | 0.68 | 0.01 | pilus assembly protein Flp/PilA - PilA | Extracellular structures |
| B488_RS06445 | 0.82 | 0.03 | biotin synthase | Coenzyme transport and metabolism |
| B488_RS06500 | 0.64 | 1.79E-06 | hypothetical protein | Function unknown |
| B488_RS06505 | 0.73 | 3.43E-04 | RNA polymerase primary sigma factor - RpoD | Transcription |
| B488_RS06595 | 0.71 | 0.01 | ATP-dependent HslUV protease, peptidase subunit HslV | Posttranslational modification, protein turnover, chaperones |
| B488_RS06630 | 0.72 | 3.31E-04 | phosphoenolpyruvate carboxykinase (ATP) | Energy production and conversion |
| B488_RS06690 | 0.67 | 3.13E-04 | acetyl-CoA carboxylase carboxyl transferase subunit beta | Lipid transport and metabolism |
| B488_RS03520 | 0.74 | 0.05 | hypothetical protein | noCOG |
| B488_RS03670 | 0.57 | 3.34E-06 | hypothetical protein | noCOG |
| B488_RS06065 | 0.74 | 0.03 | hypothetical protein | noCOG |
