# Supplementary material for: Osmotic stress induces long-term biofilm survival in Liberibacter crescens
Source: BMC Microbiol. 2022 Feb 11;22:52. doi: 10.1186/s12866-022-02453-w (PMC8832773; doi:10.1186/s12866-022-02453-w)
Supplement: Supplementary file 3 — Additional file 3: Table S3. [file 12866_2022_2453_MOESM3_ESM.docx]

**Table S3.** Up-regulated genes in *L. crescens* under osmotic stress.

| **Locus tag** | **Fold Change** | ***p*adj** | **Annotation** | **COG** |
| --- | --- | --- | --- | --- |
| B488_RS00310 | 1.35 | 0.02 | hypothetical protein | noCOG |
| B488_RS00340 | 1.31 | 0.02 | tRNA1(Val) A37 N6-methylase TrmN6 | Translation, ribosomal structure and biogenesis |
| B488_RS00385 | 1.33 | 7.34E-04 | two-component system, cell cycle response regulator | Signal transduction mechanisms |
| B488_RS00615 | 1.29 | 0.05 | RNA polymerase-binding protein DksA | Translation, ribosomal structure and biogenesis |
| B488_RS00705 | 1.29 | 0.05 | D-alanyl-D-alanine carboxypeptidase | Cell Wall Membrane/Envelope biogenesis |
| B488_RS00765 | 1.23 | 0.01 | Fe-S cluster biogenesis protein NfuA, 4Fe-4S-binding domain | Posttranslational modification, protein turnover, chaperones |
| B488_RS00895 | 1.27 | 0.01 | DNA-binding transcriptional regulator, CsgD family - VisN | Transcription |
| B488_RS01840 | 1.37 | 1.57E-04 | ATP-binding cassette, subfamily C | Intracellular trafficking, secretion, and vesicular transport |
| B488_RS01940 | 1.25 | 0.02 | hypothetical protein | noCOG |
| B488_RS02185 | 1.28 | 0.01 | large subunit ribosomal protein L31 | Translation, ribosomal structure and biogenesis |
| B488_RS02950 | 1.26 | 0.04 | glycosyl transferase, family 25 | Cell Wall Membrane/Envelope biogenesis |
| B488_RS02975 | 1.24 | 0.04 | cystine transport system permease protein | Amino acid transport and metabolism |
| B488_RS03550 | 1.36 | 4.50E-03 | F-type H+-transporting ATPase subunit a | Energy production and conversion |
| B488_RS04210 | 1.28 | 0.03 | Pimeloyl-ACP methyl ester carboxylesterase | Coenzyme transport and metabolism |
| B488_RS04230 | 1.24 | 0.02 | general L-amino acid transport system permease protein | Amino acid transport and metabolism |
| B488_RS04330 | 1.40 | 1.54E-05 | Protein of unknown function (DUF3892) | noCOG |
| B488_RS04450 | 1.49 | 4.34E-07 | hypothetical protein | Cell Wall Membrane/Envelope biogenesis |
| B488_RS04455 | 1.42 | 4.07E-04 | Cupredoxin-like domain-containing protein | noCOG |
| B488_RS04460 | 1.37 | 1.24E-04 | high-affinity iron transporter | Inorganic ion transport and metabolism |
| B488_RS04790 | 1.20 | 0.03 | outer membrane protein, multidrug efflux system - CmeC | Cell Wall Membrane/Envelope biogenesis |
| B488_RS04995 | 1.20 | 0.03 | branched-chain amino acid transport system permease protein | Amino acid transport and metabolism |
| B488_RS05400 | 1.41 | 0.01 | hypothetical protein | noCOG |
| B488_RS05465 | 1.29 | 0.05 | hypothetical protein | Function unknown |
| B488_RS05480 | 1.33 | 1.44E-03 | Methyltransferase domain-containing protein | noCOG |
| B488_RS05595 | 1.26 | 0.01 | hypothetical protein | noCOG |
| B488_RS05640 | 1.22 | 0.04 | 23S rRNA (guanosine2251-2'-O)-methyltransferase | Translation, ribosomal structure and biogenesis |
| B488_RS06210 | 1.37 | 0.04 | Fur family transcriptional regulator, zinc uptake regulator | Inorganic ion transport and metabolism |
| B488_RS06235 | 1.21 | 0.02 | tight adherence protein B - TadB | Extracellular structures |
| B488_RS06265 | 1.30 | 0.03 | prepilin peptidase CpaA | Posttranslational modification, protein turnover, chaperones |
| B488_RS06270 | 1.29 | 1.51E-04 | hypothetical protein | noCOG |
| B488_RS06285 | 1.38 | 2.68E-04 | pilus assembly protein Flp/PilA - PilA | Extracellular structures |
| B488_RS06420 | 1.22 | 0.02 | large subunit ribosomal protein L34 | Translation, ribosomal structure and biogenesis |
| B488_RS06430 | 1.17 | 0.04 | YidC/Oxa1 family membrane protein insertase - YidC | Cell Wall Membrane/Envelope biogenesis |
| B488_RS06500 | 1.28 | 1.03E-04 | hypothetical protein | Function unknown |
| B488_RS06730 | 1.27 | 0.04 | septum formation protein | Cell Wall Membrane/Envelope biogenesis |
| B488_RS06910 | 1.18 | 0.04 | Cell pole-organizing protein PopZ | Cell cycle control |
