# Supplementary material for: Osmotic stress induces long-term biofilm survival in Liberibacter crescens
Source: BMC Microbiol. 2022 Feb 11;22:52. doi: 10.1186/s12866-022-02453-w (PMC8832773; doi:10.1186/s12866-022-02453-w)
Supplement: Supplementary file 4 — Additional file 4: Table S4. [file 12866_2022_2453_MOESM4_ESM.docx]

**Table S4.** Down-regulated genes in *L. crescens* under osmotic stress.

| **Locus tag** | **Fold Change** | ***p*adj** | **Annotation** | **COG** |
| --- | --- | --- | --- | --- |
| B488_RS00155 | 0.75 | 3.16E-06 | translation initiation factor IF-2 | Translation, ribosomal structure and biogenesis |
| B488_RS00190 | 0.78 | 0.01 | 2,3,4,5-tetrahydropyridine-2-carboxylate N-succinyltransferase | Amino acid transport and metabolism |
| B488_RS00200 | 0.82 | 4.21E-03 | small subunit ribosomal protein S2 | Translation, ribosomal structure and biogenesis |
| B488_RS00205 | 0.84 | 0.03 | elongation factor Ts | Translation, ribosomal structure and biogenesis |
| B488_RS00235 | 0.86 | 0.03 | Beta-barrel assembly machine subunit BamA | Cell Wall Membrane/Envelope biogenesis |
| B488_RS00290 | 0.76 | 9.60E-04 | hypothetical protein | General function prediction only |
| B488_RS06760 | 0.77 | 1.52E-03 | BA14K-like protein | noCOG |
| B488_RS00380 | 0.82 | 0.04 | two-component system, cell cycle response regulator DivK | Signal transduction mechanisms |
| B488_RS00425 | 0.76 | 4.63E-03 | translation initiation factor IF-1 | Translation, ribosomal structure and biogenesis |
| B488_RS00435 | 0.65 | 7.82E-07 | hypothetical protein | Replication, recombination and repair |
| B488_RS00470 | 0.75 | 2.39E-06 | branched-chain amino acid transport system substrate-binding protein | Amino acid transport and metabolism |
| B488_RS00480 | 0.66 | 4.24E-10 | branched-chain amino acid transport system substrate-binding protein | Amino acid transport and metabolism |
| B488_RS00540 | 0.83 | 0.01 | TIGR02300 family protein | Function unknown |
| B488_RS00545 | 0.80 | 0.01 | 3-phosphoshikimate 1-carboxyvinyltransferase | Amino acid transport and metabolism |
| B488_RS00555 | 0.86 | 0.02 | small subunit ribosomal protein S1 | Translation, ribosomal structure and biogenesis |
| B488_RS01075 | 0.81 | 0.03 | ferredoxin--NADP+ reductase | Energy production and conversion |
| B488_RS01205 | 0.85 | 0.02 | leucyl aminopeptidase | Amino acid transport and metabolism |
| B488_RS01265 | 0.60 | 2.47E-20 | chaperonin GroES- GroES | Posttranslational modification, protein turnover, chaperones |
| B488_RS01270 | 0.72 | 1.54E-09 | chaperonin GroEL - GroEL | Posttranslational modification, protein turnover, chaperones |
| B488_RS01410 | 0.82 | 0.01 | ATP-dependent Clp protease ATP-binding subunit ClpB - ClpB | Posttranslational modification, protein turnover, chaperones |
| B488_RS01455 | 0.83 | 0.01 | Invasion protein IalB, involved in pathogenesis | General function prediction only |
| B488_RS01515 | 0.79 | 3.12E-04 | peptidyl-prolyl cis-trans isomerase D - SurA | Posttranslational modification, protein turnover, chaperones |
| B488_RS01600 | 0.78 | 3.49E-04 | small subunit ribosomal protein S6 | Translation, ribosomal structure and biogenesis |
| B488_RS01620 | 0.76 | 7.00E-06 | peptide/nickel transport system substrate-binding protein | Amino acid transport and metabolism |
| B488_RS01645 | 0.64 | 2.52E-13 | peptidyl-prolyl cis-trans isomerase C - PrsA | Posttranslational modification, protein turnover, chaperones |
| B488_RS01650 | 0.80 | 0.01 | glutamate N-acetyltransferase | Amino acid transport and metabolism |
| B488_RS01660 | 0.72 | 1.38E-03 | hypothetical protein | noCOG |
| B488_RS01675 | 0.76 | 1.61E-05 | carboxyl-terminal processing protease – S41 protease | Posttranslational modification, protein turnover, chaperones |
| B488_RS01825 | 0.80 | 0.01 | NAD(P)H dehydrogenase (quinone) | General function prediction only |
| B488_RS01885 | 0.84 | 0.04 | single-strand DNA-binding protein | Replication, recombination and repair |
| B488_RS01985 | 0.82 | 2.98E-03 | elongation factor G | Translation, ribosomal structure and biogenesis |
| B488_RS02020 | 0.65 | 2.50E-10 | small subunit ribosomal protein S19 | Translation, ribosomal structure and biogenesis |
| B488_RS02030 | 0.76 | 1.45E-04 | small subunit ribosomal protein S3 | Translation, ribosomal structure and biogenesis |
| B488_RS02040 | 0.82 | 0.03 | large subunit ribosomal protein L29 | Translation, ribosomal structure and biogenesis |
| B488_RS02045 | 0.73 | 4.40E-05 | small subunit ribosomal protein S17 | Translation, ribosomal structure and biogenesis |
| B488_RS02060 | 0.85 | 0.03 | large subunit ribosomal protein L5 | Translation, ribosomal structure and biogenesis |
| B488_RS02070 | 0.84 | 0.03 | small subunit ribosomal protein S8 | Translation, ribosomal structure and biogenesis |
| B488_RS02095 | 0.74 | 1.91E-06 | large subunit ribosomal protein L15 | Translation, ribosomal structure and biogenesis |
| B488_RS02110 | 0.73 | 1.10E-05 | small subunit ribosomal protein S13 | Translation, ribosomal structure and biogenesis |
| B488_RS02225 | 0.80 | 0.01 | Uncharacterized conserved protein, DUF2336 family | Function unknown |
| B488_RS02335 | 0.80 | 0.04 | hypothetical protein | noCOG |
| B488_RS02590 | 0.82 | 0.02 | phosphoribosylaminoimidazole-succinocarboxamide synthase | Nucleotide transport and metabolism |
| B488_RS02760 | 0.85 | 0.02 | ATP-binding cassette protein, ChvD family | General function prediction only |
| B488_RS02820 | 0.83 | 0.02 | Regulator of protease activity HflC, stomatin/prohibitin superfamily | Posttranslational modification, protein turnover, chaperones |
| B488_RS02845 | 0.83 | 0.02 | DNA-binding transcriptional response regulator, NtrC family, contains REC, AAA-type ATPase, and a Fis-type DNA-binding domains – NtrC/AtoC | Signal transduction mechanisms |
| B488_RS02875 | 0.81 | 0.01 | cysteine synthase A | Amino acid transport and metabolism |
| B488_RS02905 | 0.78 | 1.36E-03 | cell division protein FtsA - FtsA | Cell cycle control |
| B488_RS02910 | 0.69 | 1.12E-09 | cell division protein FtsZ – FtsZ | Cell cycle control |
| B488_RS03180 | 0.76 | 0.02 | ATP-grasp domain-containing protein | noCOG |
| B488_RS03230 | 0.72 | 1.42E-07 | pilus assembly protein Flp/PilA | Extracellular structures |
| B488_RS03280 | 0.82 | 0.02 | Glycosyl transferase family 2 | noCOG |
| B488_RS03575 | 0.83 | 0.01 | dihydrolipoamide dehydrogenase - LdpC | Energy production and conversion |
| B488_RS03580 | 0.82 | 0.01 | 2-oxoglutarate dehydrogenase E2 component - SucA | Energy production and conversion |
| B488_RS03595 | 0.82 | 0.01 | succinyl-CoA synthetase (ADP-forming) beta subunit | Energy production and conversion |
| B488_RS03815 | 0.76 | 2.00E-04 | zinc/manganese transport system substrate-binding protein | Inorganic ion transport and metabolism |
| B488_RS03945 | 0.75 | 1.34E-03 | Excinuclease ABC subunit B | Replication, recombination and repair |
| B488_RS04010 | 0.75 | 0.02 | Predicted N-acetyltransferase YhbS | General function prediction only |
| B488_RS04045 | 0.82 | 0.01 | large subunit ribosomal protein L7/L12 | Translation, ribosomal structure and biogenesis |
| B488_RS04100 | 0.85 | 0.02 | D-3-phosphoglycerate dehydrogenase | Coenzyme transport and metabolism |
| B488_RS04170 | 0.83 | 0.03 | Fe-S cluster assembly protein SufD | Posttranslational modification, protein turnover, chaperones |
| B488_RS04370 | 0.77 | 3.70E-03 | DNA-binding protein HU-beta | Replication, recombination and repair |
| B488_RS04700 | 0.79 | 3.62E-03 | acyl carrier protein | Lipid transport and metabolism |
| B488_RS04710 | 0.85 | 0.03 | aspartyl/glutamyl-tRNA(Asn/Gln) amidotransferase subunit A | Translation, ribosomal structure and biogenesis |
| B488_RS04715 | 0.71 | 4.03E-05 | aspartyl/glutamyl-tRNA(Asn/Gln) amidotransferase subunit C | Translation, ribosomal structure and biogenesis |
| B488_RS04900 | 0.80 | 5.08E-04 | 3-oxoacyl-[acyl-carrier-protein] synthase II | Lipid transport and metabolism |
| B488_RS04905 | 0.78 | 3.12E-04 | acyl carrier protein | Lipid transport and metabolism |
| B488_RS04910 | 0.62 | 1.89E-15 | 3-oxoacyl-[acyl-carrier-protein] reductase | Lipid transport and metabolism |
| B488_RS04920 | 0.63 | 9.44E-04 | manganese/iron transport system substrate-binding protein | Inorganic ion transport and metabolism |
| B488_RS04925 | 0.58 | 4.82E-05 | manganese/iron transport system ATP-binding protein | Inorganic ion transport and metabolism |
| B488_RS04930 | 0.68 | 0.01 | manganese/iron transport system permease protein | Inorganic ion transport and metabolism |
| B488_RS04935 | 0.75 | 0.01 | manganese/iron transport system permease protein | Inorganic ion transport and metabolism |
| B488_RS04955 | 0.74 | 1.37E-06 | GTP-binding protein | Signal transduction mechanisms |
| B488_RS04960 | 0.79 | 1.03E-03 | argininosuccinate synthase | Amino acid transport and metabolism |
| B488_RS04980 | 0.76 | 6.40E-05 | branched-chain amino acid transport system ATP-binding protein | Amino acid transport and metabolism |
| B488_RS05010 | 0.73 | 3.16E-06 | cell cycle transcriptional regulator CtrA | Signal transduction mechanisms |
| B488_RS05025 | 0.83 | 0.03 | hypothetical protein | Function unknown |
| B488_RS05115 | 0.84 | 0.02 | Peroxiredoxin | Posttranslational modification, protein turnover, chaperones |
| B488_RS05150 | 0.80 | 0.01 | exopolyphosphatase / guanosine-5'-triphosphate,3'-diphosphate pyrophosphatase | Nucleotide transport and metabolism |
| B488_RS05160 | 0.83 | 3.96E-03 | Porin subfamily protein | noCOG |
| B488_RS05190 | 0.68 | 2.64E-10 | large subunit ribosomal protein L35 | Translation, ribosomal structure and biogenesis |
| B488_RS05195 | 0.84 | 0.01 | translation initiation factor IF-3 | Translation, ribosomal structure and biogenesis |
| B488_RS05275 | 0.81 | 0.03 | NusB antitermination factor | Transcription |
| B488_RS05280 | 0.80 | 0.02 | 6,7-dimethyl-8-ribityllumazine synthase | Coenzyme transport and metabolism |
| B488_RS05285 | 0.82 | 0.04 | riboflavin synthase alpha chain | Coenzyme transport and metabolism |
| B488_RS05360 | 0.71 | 6.07E-09 | pyruvate dehydrogenase E1 component beta subunit | Energy production and conversion |
| B488_RS05485 | 0.75 | 0.03 | hydroxyacylglutathione hydrolase | General function prediction only |
| B488_RS05515 | 0.79 | 0.03 | shikimate kinase - AroK | Amino acid transport and metabolism |
| B488_RS05660 | 0.83 | 0.01 | Glycine zipper | noCOG |
| B488_RS06050 | 0.84 | 0.03 | phosphatidylglycerol:prolipoprotein diacylglycerol transferase | Cell Wall Membrane/Envelope biogenesis |
| B488_RS06080 | 0.81 | 5.67E-04 | serine protease Do - HtrA | Posttranslational modification, protein turnover, chaperones |
| B488_RS06325 | 0.78 | 0.02 | lipopolysaccharide export system ATP-binding protein | Cell Wall Membrane/Envelope biogenesis |
| B488_RS06470 | 0.72 | 1.37E-06 | peroxiredoxin, Ohr subfamily | Defense Mechanisms |
| B488_RS06690 | 0.75 | 3.12E-04 | acetyl-CoA carboxylase carboxyl transferase subunit beta | Lipid transport and metabolism |
| B488_RS06725 | 0.83 | 0.05 | shikimate dehydrogenase - AroE | Amino acid transport and metabolism |
| B488_RS00535 | 0.75 | 3.70E-03 | hypothetical protein | noCOG |
| B488_RS04075 | 0.76 | 0.02 | hypothetical protein | noCOG |
