# Supplementary material for: Osmotic stress induces long-term biofilm survival in Liberibacter crescens
Source: BMC Microbiol. 2022 Feb 11;22:52. doi: 10.1186/s12866-022-02453-w (PMC8832773; doi:10.1186/s12866-022-02453-w)
Supplement: Supplementary file 5 — Additional file 5: Table S5. [file 12866_2022_2453_MOESM5_ESM.docx]

**Table S5.**  Up-regulated genes in *L. crescens* under DMSO stress.

| **Locus tag** | **Fold Change** | ***p*adj** | **Annotation** | **COG** |
| --- | --- | --- | --- | --- |
| B488_RS01660 | 1.41 | 0.01 | hypothetical protein | noCOG |
| B488_RS01870 | 1.35 | 0.02 | glutamine synthetase | Amino acid transport and metabolism |
| B488_RS02185 | 1.66 | 4.33E-06 | large subunit ribosomal protein L31 | Translation, ribosomal structure and biogenesis |
| B488_RS03150 | 1.49 | 1.38E-04 | cold shock protein (beta-ribbon, CspA family) | Transcription |
| B488_RS03355 | 1.41 | 4.01E-03 | DNA-directed RNA polymerase subunit omega | Transcription |
| B488_RS03990 | 1.34 | 0.01 | hypothetical protein | noCOG |
| B488_RS03995 | 1.36 | 0.02 | glutathione S-transferase | Posttranslational modification, protein turnover, chaperones |
| B488_RS04140 | 1.42 | 4.01E-03 | large subunit ribosomal protein L36 | Translation, ribosomal structure and biogenesis |
| B488_RS04385 | 1.37 | 0.01 | ATP-dependent Clp protease, protease subunit | Posttranslational modification, protein turnover, chaperones |
| B488_RS04390 | 1.46 | 3.25E-04 | large subunit ribosomal protein L13 | Translation, ribosomal structure and biogenesis |
| B488_RS04720 | 1.40 | 4.24E-03 | L-ascorbate metabolism protein UlaG, beta-lactamase superfamily | Carbohydrate transport and metabolism |
| B488_RS05795 | 1.44 | 2.19E-03 | small subunit ribosomal protein S20 | Translation, ribosomal structure and biogenesis |
| B488_RS06065 | 1.37 | 0.02 | hypothetical protein | noCOG |
| B488_RS06150 | 1.38 | 4.01E-03 | phosphate transport system substrate-binding protein | Inorganic ion transport and metabolism |
| B488_RS06215 | 1.29 | 0.04 | ferritin | Inorganic ion transport and metabolism |
| B488_RS06285 | 1.50 | 2.09E-03 | pilus assembly protein Flp/PilA | Extracellular structures |
| B488_RS06760 | 1.35 | 0.03 | BA14K-like protein | noCOG |
