# Supplementary material for: Osmotic stress induces long-term biofilm survival in Liberibacter crescens
Source: BMC Microbiol. 2022 Feb 11;22:52. doi: 10.1186/s12866-022-02453-w (PMC8832773; doi:10.1186/s12866-022-02453-w)
Supplement: Supplementary file 6 — Additional file 6: Table S6. [file 12866_2022_2453_MOESM6_ESM.docx]

**Table S6.** Down-regulated genes in *L. crescens* under DMSO stress.

| **Locus tag** | **Fold Change** | ***p*adj** | **Annotation** | **COG** |
| --- | --- | --- | --- | --- |
| B488_RS02640 | 0.73 | 0.00E+00 | dUTP pyrophosphatase | Defense Mechanisms |
| B488_RS04570 | 0.76 | 0.05 | flagellin | Cell Motility |
| B488_RS04740 | 0.74 | 0.02 | ATP-binding protein involved in chromosome partitioning | Cell cycle control |
| B488_RS06555 | 0.71 | 0.00E+00 | ATP-binding protein involved in chromosome partitioning | Function unknown |
| B488_RS06800 | 0.72 | 0.03 | hypothetical protein | noCOG |
