# Supplementary material for: Osmotic stress induces long-term biofilm survival in Liberibacter crescens
Source: BMC Microbiol. 2022 Feb 11;22:52. doi: 10.1186/s12866-022-02453-w (PMC8832773; doi:10.1186/s12866-022-02453-w)
Supplement: Supplementary file 7 — Additional file 7: Table S7. [file 12866_2022_2453_MOESM7_ESM.docx]

**Table S7.** Summary of the differentially expressed (DE) genes in each stress condition (padj ≤ 0.05).

| Condition | Total DE genes | Up-regulated | Down-regulated |
| --- | --- | --- | --- |
| Heat | 240 | 117 | 123 |
| Percent of genome | (17.7 %)^1^ | (8.6 %)^1^ | (9.1 %)^1^ |
| Osmotic | 132 | 36 | 96 |
| Percent of genome | (9.7 %)^1^ | (2.6 %)^1^ | (7.1 %)^1^ |
| DMSO | 22 | 17 | 5 |
| Percent of genome | (1.6 %)^1^ | (1.3 %)^1^ | (0.4 %)^1^ |

^1^The percentages were calculated using 1355 gene locus extracted from NCBI database.
