# Supplementary figures and images for: Osmotic stress induces long-term biofilm survival in Liberibacter crescens
Source: BMC Microbiol. 2022 Feb 11;22:52. doi: 10.1186/s12866-022-02453-w (PMC8832773; doi:10.1186/s12866-022-02453-w)

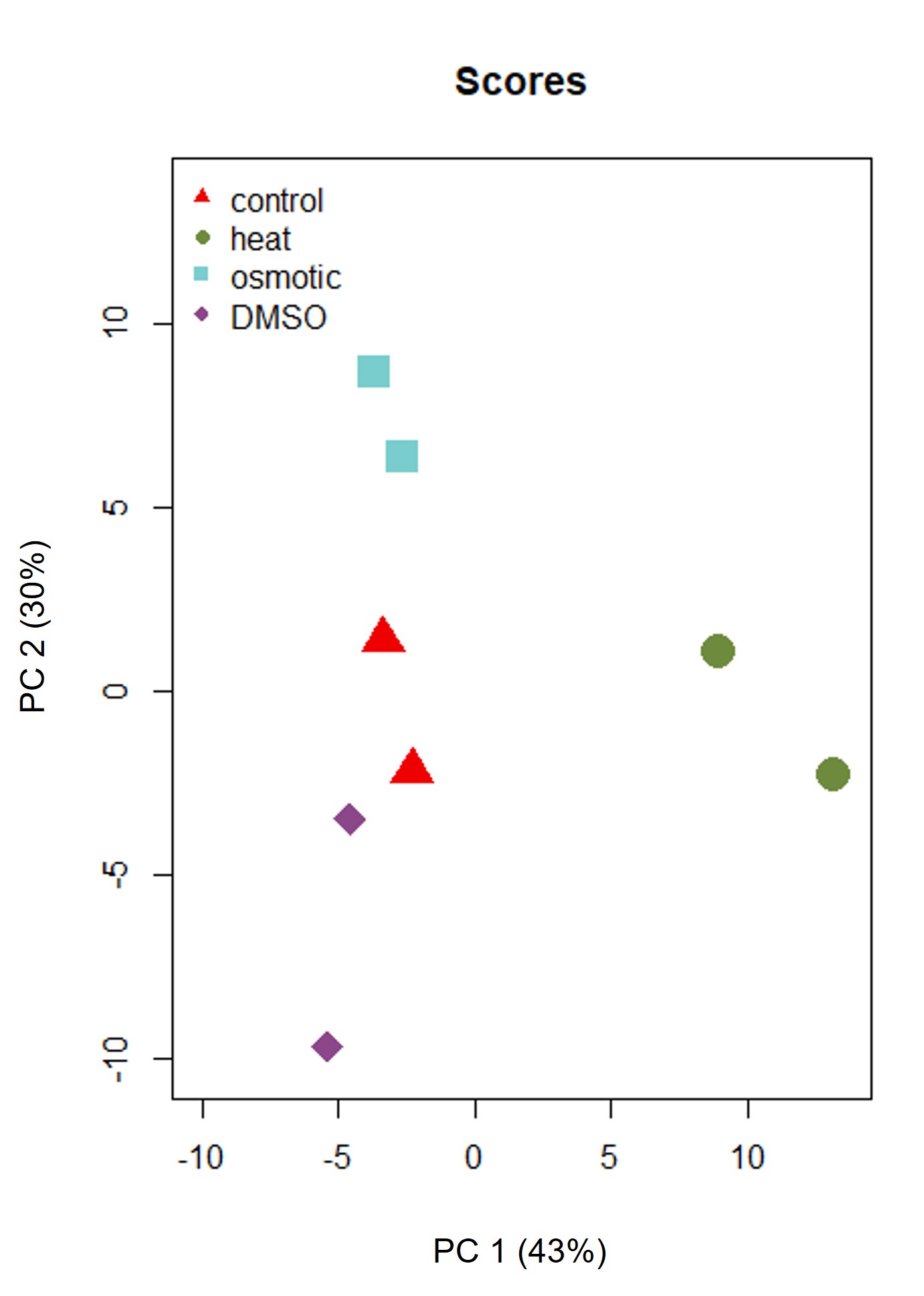

Supplement: Supplementary file 8 — Additional file 8: Figure S1. [file 12866_2022_2453_MOESM8_ESM.jpg]

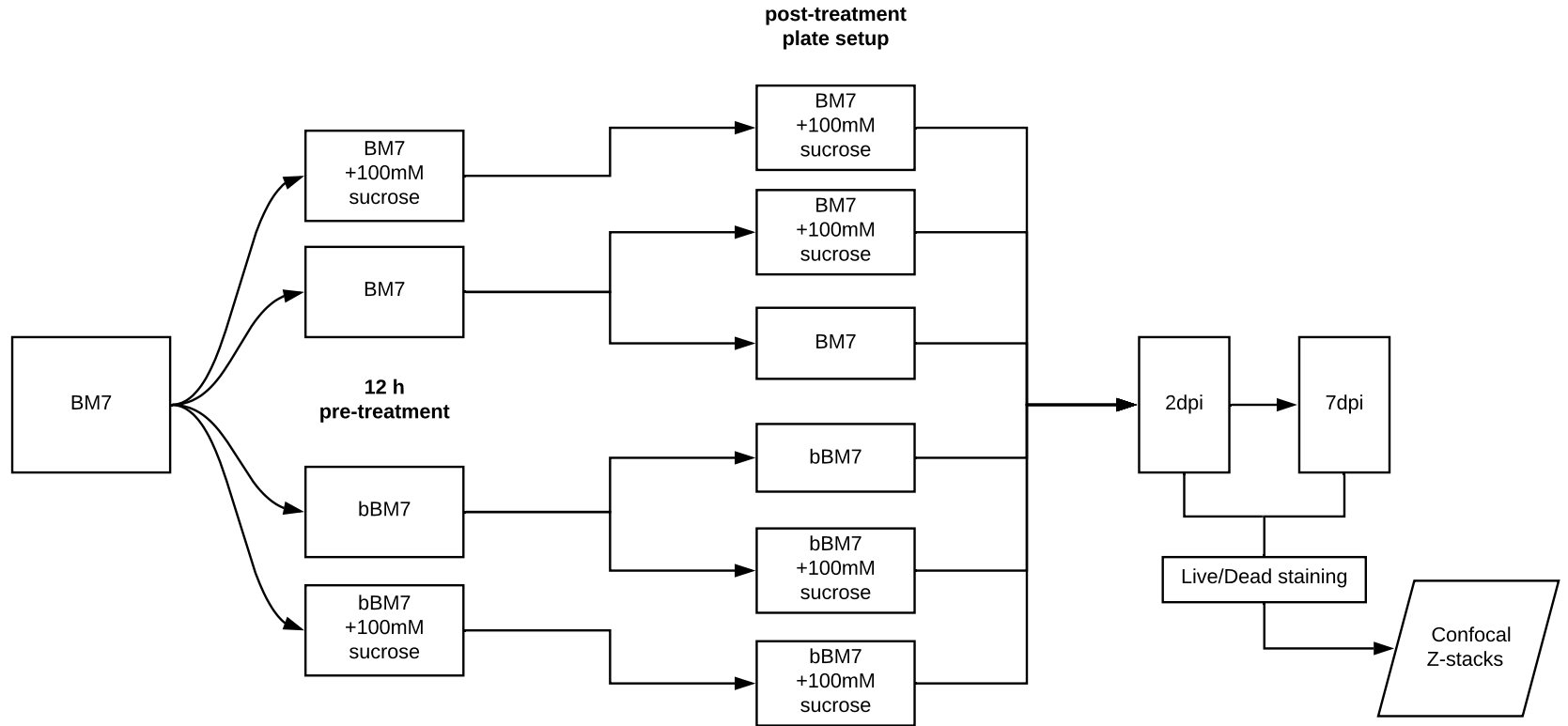

Supplement: Supplementary file 10 — Additional file 10: Figure S2. [file 12866_2022_2453_MOESM10_ESM.pdf]
