# Supplementary material for: Osmotic stress induces long-term biofilm survival in Liberibacter crescens
Source: BMC Microbiol. 2022 Feb 11;22:52. doi: 10.1186/s12866-022-02453-w (PMC8832773; doi:10.1186/s12866-022-02453-w)
Supplement: Supplementary file 9 — Additional file 9: Table S8. [file 12866_2022_2453_MOESM9_ESM.docx]

**Table S8.** Probability distribution of the number of up- and down-regulated genes from *L. crescens* BT-1. Numbers highlighted in grey are the significant (*p*≤0.05) COG categories identified by a hypergeometric test, where numbers in bold are affected COG categories determined by percent change from the control.

|  | **Up-regulated** | | | **Down-regulated** | | |
| --- | --- | --- | --- | --- | --- | --- |
| **COG** | Heat | Osmotic | DMSO | Heat | Osmotic | DMSO |
| Energy production and conversion (C) | 0.15 | 0.29 | 0.40 | 0.12 | 0.19 | 0.76 |
| Cell cycle control (D) | 0.07 | 0.33 | 0.77 | 0.28 | 0.26 | 0.07 |
| Amino acid transport and metabolism (E) | **0.03** | 0.24 | 0.36 | 0.00 | **0.03** | 0.66 |
| Nucleotide transport and metabolism (F) | 0.03 | 0.34 | 0.60 | 0.22 | 0.24 | 0.86 |
| Carbohydrate transport and metabolism (G) | 0.11 | 0.35 | 0.31 | 0.19 | 0.06 | 0.87 |
| Coenzyme transport and metabolism (H) | 0.14 | 0.30 | 0.41 | 0.11 | 0.15 | 0.77 |
| Lipid transport and metabolism (I) | 0.22 | 0.32 | 0.58 | 0.16 | 0.10 | 0.85 |
| Translation, ribosomal structure, and biogenesis (J) | 0.01 | 0.16 | 0.07 | **<0.01** | **<0.01** | 0.57 |
| Transcription (K) | 0.23 | 0.17 | 0.06 | **0.02** | 0.21 | 0.11 |
| Replication, recombination, and repair (L) | 0.13 | 0.14 | 0.40 | 0.15 | 0.18 | 0.76 |
| Cell Wall Membrane/Envelope biogenesis (M) | 0.12 | **0.02** | 0.33 | 0.13 | 0.08 | 0.72 |
| Cell Motility (N) | 0.15 | 0.40 | 0.65 | 0.18 | 0.08 | 0.11 |
| Posttranslational modification, protein turnover, chaperones (O) | 0.16 | 0.27 | 0.14 | 0.08 | **0.01** | 0.80 |
| Inorganic ion transport and metabolism (P) | **0.01** | 0.37 | **0.04** | 0.18 | **0.04** | 0.90 |
| Secondary metabolites biosynthesis, transport, and catabolism (Q) | 0.08 | 0.85 | 0.93 | 0.56 | 0.64 | 0.98 |
| General function prediction only (R) | 0.22 | 0.37 | 0.63 | 0.20 | **0.03** | 0.87 |
| Function unknown (S) | 0.13 | 0.24 | 0.54 | 0.13 | 0.23 | 0.15 |
| Signal transduction mechanisms (T) | 0.24 | 0.37 | 0.66 | 0.17 | 0.12 | 0.89 |
| Intracellular trafficking, secretion, and vesicular transport (U) | 0.16 | 0.32 | 0.78 | 0.28 | 0.23 | 0.93 |
| Defense Mechanisms (V) | **<0.01** | 0.65 | 0.82 | 0.22 | 0.38 | 0.06 |
| Extracellular structures (W) | 0.23 | **0.05** | 0.17 | 0.35 | 0.38 | 0.94 |
| not in COG | 0.09 | 0.15 | 0.22 | <0.01 | <0.01 | 0.24 |
