# Supplementary material for: Osmotic stress induces long-term biofilm survival in Liberibacter crescens
Source: BMC Microbiol. 2022 Feb 11;22:52. doi: 10.1186/s12866-022-02453-w (PMC8832773; doi:10.1186/s12866-022-02453-w)
Supplement: Supplementary file 11 — Additional file 11: Table S9. [file 12866_2022_2453_MOESM11_ESM.docx]

**Table S9.** Coefficient of determination (R^2^) values given by the correlation tests between significant DE genes of *L*. *crescens* under different stress conditions or antimicrobial treatments.

| Correlation test | R^2^ |
| --- | --- |
| Benzbromarone vs Phloretin | 0.85 |
| Benzbromarone vs Tolfenamic Acid | 0.09 |
| Phloretin vs Tolfenamic Acid | 0.06 |
| Benzbromarone vs Heat | 0.05 |
| Phloretin vs Heat | 0.03 |
| Tolfenamic Acid vs Heat | 0.05 |
| Osmotic Stress vs Heat | 0.04 |
| Phloretin vs Osmotic Stress | <0.01 |
| Tolfenamic acid vs Osmotic Stress | 0.37 |
| Benzbromarone vs Osmotic Stress | <0.01 |
